# From VIB- to VB-Group Transition Metal Disulfides: Structure Engineering Modulation for Superior Electromagnetic Wave Absorption

**DOI:** 10.1007/s40820-023-01247-7

**Published:** 2023-11-23

**Authors:** Junye Cheng, Yongheng Jin, Jinghan Zhao, Qi Jing, Bailong Gu, Jialiang Wei, Shenghui Yi, Mingming Li, Wanli Nie, Qinghua Qin, Deqing Zhang, Guangping Zheng, Renchao Che

**Affiliations:** 1 Department of Materials Science, Shenzhen MSU-BIT University, Shenzhen, 517182 People’s Republic of China; 2 School of Materials Science and Engineering, Beijing Institute of Technology, Beijing, 100081 People’s Republic of China; 3 School of Materials Science and Engineering, Qiqihar University, Qiqihar, 161006 People’s Republic of China; 4 Department of Mechanical Engineering, Hong Kong Polytechnic University, Hung Hom, Kowloon, Hong Kong, People’s Republic of China; 5 Laboratory of Advanced Materials, Shanghai Key Lab of Molecular Catalysis and Innovative Materials, Fudan University, Shanghai, 200438 People’s Republic of China; 6 Zhejiang Laboratory, Hangzhou, 311100 People’s Republic of China

**Keywords:** Transition metal disulfides, Electromagnetic wave absorption, Impedance matching, Structure engineering modulation

## Abstract

The laminated transition metal disulfides (TMDs), which are well known as typical two-dimensional (2D) semiconductive materials, possess a unique layered structure, leading to their wide-spread applications in various fields, such as catalysis, energy storage, sensing, etc. In recent years, a lot of research work on TMDs based functional materials in the fields of electromagnetic wave absorption (EMA) has been carried out. Therefore, it is of great significance to elaborate the influence of TMDs on EMA in time to speed up the application. In this review, recent advances in the development of electromagnetic wave (EMW) absorbers based on TMDs, ranging from the VIB group to the VB group are summarized. Their compositions, microstructures, electronic properties, and synthesis methods are presented in detail. Particularly, the modulation of structure engineering from the aspects of heterostructures, defects, morphologies and phases are systematically summarized, focusing on optimizing impedance matching and increasing dielectric and magnetic losses in the EMA materials with tunable EMW absorption performance. Milestones as well as the challenges are also identified to guide the design of new TMDs based dielectric EMA materials with high performance.

## Highlights

- A systematic summary of current research trends in the development of transition metal disulfides (TMDs) electromagnetic wave (EMW) absorption materials.
- In-depth comparisons on the structures, preparation methods, application merits of VIB- and VB-group TMDs.
- Structure engineering modulation of TMDs in achieving superior EMW absorption is outlined from the viewpoints of heterostructures, defects, morphologies, and phases.
- Exclusive insights into the challenges, strategies, and opportunities in the design of EMW absorption materials with outstanding performance are provided.

## Graphical abstract

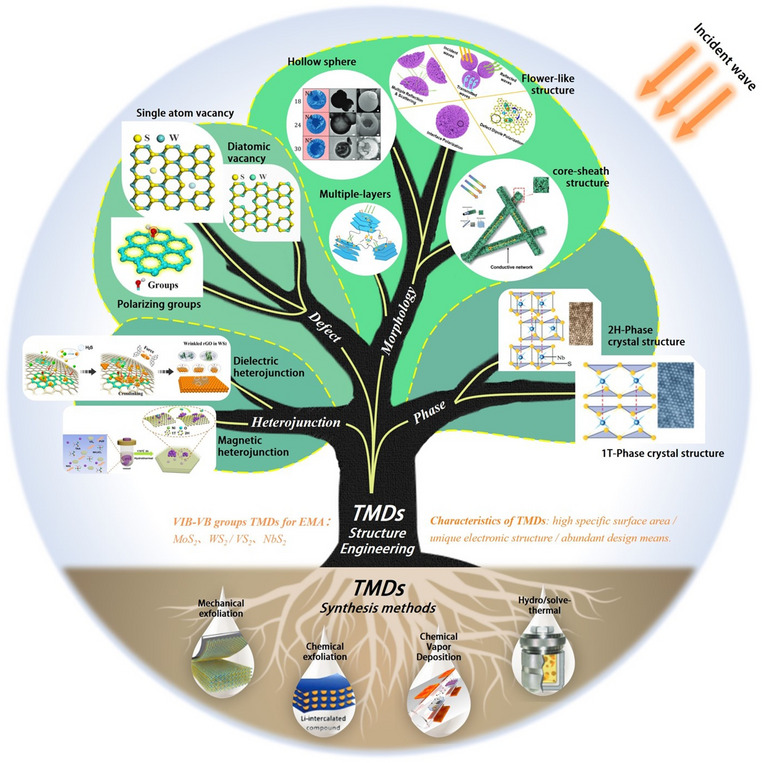

## Introduction

The ongoing technological revolution, marked by the advances in artificial intelligence, wireless communication, and the metaverse, is fundamentally reshaping our society with greater intelligence, information-centricity, and convenience [1–4]. Concurrently, the widespread application of 5G technology and the pervasive use of electronic devices have led to an alarming increase in electromagnetic pollution. The surge in electromagnetic pollution poses significant threats to both national security and human health [5–10]. Consequently, there is a growing demand for electromagnetic wave absorption materials that can effectively mitigate the adverse effects of electromagnetic radiation. Research in this field is dedicated to developing protective and stealth solutions that can address the escalating complexity of the electromagnetic environment and the evolving technological demands [11, 12]. electromagnetic wave absorption (EMA) materials have widespread applications in everyday life due to their ability to absorb, reflect, or scatter electromagnetic waves, providing diverse functionalities. In electronic devices and medical equipment, these materials prevent electromagnetic radiation leakage and interference, ensuring proper functionality and safety. They can reduce radar cross sections in military applications, making aircraft, ships, and vehicles less detectable, and they improve the performance of communication systems by minimizing interference and beamforming issues. They are also employed in radiation-protective clothing to mitigate the impact of electromagnetic radiation, especially in professionals involving in prolonged exposure to electronic devices. In summary, EMA materials significantly enhance the performance, safety, and efficiency of electronic devices, communication systems, and medical applications in modern life. With rapid technological advancements, EMA materials continue to expand their scope and impact across various domains.

Transition metal disulfides (TMDs) are typical 2D materials that have been extensively applied to batteries, supercapacitors, electrocatalysis, hydrogen evolution, sensors, etc. [13]. Due to their remarkable physical and chemical attributes, TMDs have recently been investigated in the area of electromagnetic wave (EMW) absorption and have shown promising applications. Despite the inclusion of metallic elements in TMDs, they have some unique physicochemical properties favorable to the absorption of electromagnetic waves, as compared to conventional metal-based wave absorbing materials. These superior characteristics can be summarized as follows: Firstly, the high-ratio surface area brought by the laminated structure makes incident electromagnetic waves to be reflected and absorbed multiple times inside the EMA material and produces a strong interfacial polarization, which enhances the attenuation ability of the electromagnetic wave. Secondly, the electronic structure of TMDs with different crystal structures is different, especially the band gap, which greatly influences the conductivity of the EMA material. As we all know, the smaller the bandgap of a material, the higher the conductivity, the stronger the dielectric loss, and thus the more electromagnetic energy is attenuated. Thirdly, there are a variety of design routes for the preparation of transition metal disulfides, mainly including top to down methods (e.g., mechanical stripping, chemical stripping) and bottom to up methods (e.g., chemical vapor deposition, hydro/solve-thermal synthesis), which have great potential for application in the domain of EMW absorption [14].

With the aim of further improving the absorbing performance of EMA materials and exploring their structure–property relationships, extensive research on the effects of material morphology, phase structure, and heterogeneous structures on electromagnetic wave attenuation properties are carried out. The construction of materials morphologies with multiple interfaces, such as hierarchical structures, hollow spheres, core–shell structures, and egg-shell structures, is also considered to be an effectual approach to enhance the EMW absorption capacity of the material. Materials with such morphological characteristics can extend the transmission paths of the incident electromagnetic wave because of the multiple interfaces in the materials which enable incident electromagnetic waves to be reflected and absorbed multiple times. In addition, TMDs have a "sandwich"-like atomic structure, with a layer of Mo atoms sandwiched between two layers of S atoms [14]. While atoms in an individual layer are linked by intense chemical bonds, the layers are linked by weaker van der Waals (vdW) forces. TMDs with this special atomic structure commonly exhibit three types of different crystals phases, namely 1 T, 2H, and 3R. At ambient conditions, TMDs are in a 2H phase, which cannot achieve strong electromagnetic wave absorption due to its low conductivity [15]–[19]. Thus, TMDs with a 1 T phase or 1 T/2H dual phase is designed to improve their conductivity and achieve excellent electromagnetic wave attenuation performance. Alternatively, excellent EMW absorption properties of TMDs can be achieved by constructing heterogeneous interfaces in the EMA materials. The synergetic effect, especially the dielectric and magnetic synergistic effect, can make full use of the dielectric loss and magnetic loss to attenuate the incident electromagnetic wave to the maximum extent, and could be one of the most efficient methods to improve the EMW absorption capability of TMDs.

In this review, we first introduce the composition, crystal structure, and electronic properties of TMDs, and clarify the relationship between microstructure and electromagnetic properties. Then, various synthesis routes of transition metal disulfides are described, and the applications of those methods in preparing different TMDs are summarized and tabulated. Furthermore, a large number of cases of structural engineering that are related to the regulations on the EMW absorption capability of TMDs and the principles behind them are elaborated, emphasizing the significance of morphology, phase structure, defect, synergy between dielectric and magnetic losses in improving EMW absorption characteristics. Finally, by comparing publications on the application of TMDs in the area of EMW absorption, we summarize some challenges remained, and the future research directions for developing transition metal disulfides in the area of EMW absorption are proposed.

## EMW Absorption and Attenuation Mechanisms

When EMWs hit the material surface, they can be categorized into three parts: reflected waves, dissipated energy, and transmitted waves [20]. According to the plane wave model, all transmitted waves are eventually reflected at the absorber-metal surface [21]. In this scenario, effective EMW absorption materials aim to minimize the reflection of waves while maximizing the dissipation of energy. The absorption performance of EMA materials primarily relies on two key factors, i.e., impedance matching and attenuation capacity [5]. Impedance matching ensures that incident electromagnetic waves are directed into the absorbers without surface reflection, which is a fundamental requirement in EMA materials design. Once inside the absorber, the EMW energy can then be either dissipated or converted into other forms of energy, primarily influenced by dielectric and magnetic losses.

The EMW absorption performance hinges on two critical aspects: the attenuation constant (α) and reflection loss (RL) values that are defined and calculated by Eqs. (1) and (2), respectively. A higher α value signifies more effective EMW energy dissipation, while smaller RL values indicate better EMW absorption capacity, especially when RL is less than -10 dB, denoting the effective absorption bandwidth (EAB). These characteristics are fundamentally influenced by the material's electromagnetic parameters, specifically the complex permittivity (ε_r_ = ε'—jε'') and complex permeability (μ_r_ = μ'—jμ''). These parameters, which encapsulate energy storage (real part) and dissipation (imaginary part), are vital in assessing EMW absorption performance using transmission line theory:

1 $$ RL = 20\lg \left| {\frac{{Z_{in} - Z_{0} }}{{Z_{in} + Z_{0} }}} \right| $$

2 $$ \alpha = \left( {\sqrt 2 \pi f/c} \right) \times \sqrt {\left( {\mu^{\prime\prime}\varepsilon^{\prime\prime} - \mu^{\prime}\varepsilon^{\prime}} \right) + \sqrt {\left( {\mu^{\prime\prime}\varepsilon^{\prime\prime} - \mu^{\prime}\varepsilon^{\prime}} \right)^{2} + \left( {\varepsilon^{\prime}\mu^{\prime\prime} - \varepsilon^{\prime\prime}\mu^{\prime}} \right)^{2} } } $$

In summary, an excellent TMDs-based EMA material should firstly satisfy the condition of impedance matching, according to the impedance matching formula (Eq. 3), i.e., the mutual coordination of dielectric constant and magnetic permeability. Secondly, the unique phase structure of the TMDs enables it to change from a semiconductor to a conductor, which greatly improves the tunability of the electrical conductivity and facilitates the enhancement of the conductive losses. According to the free electron theory, the larger the conductivity (σ), the larger the dielectric constant (εr), and theoretically the stronger the attenuation of incident electromagnetic waves that can be performed. In addition, TMDs, as lamellar materials, have intrinsic large specific surface areas, and if they are compounded with other materials to form heterojunctions, the interfaces within the complexes will be enlarged and polarization relaxation will occur at the interfaces. Structural defects will be created in the synthesis or post-processing of the TMDs, and the resultant large number of dipoles will be polarized under varying applied electromagnetic fields and thus attenuate the electromagnetic energy. On the other hand, TMDs with a hierarchical structural morphology can lengthen the transmission path of electromagnetic waves inside the material, thereby enhancing their scattering and reflection and increasing electromagnetic losses. Therefore, to obtain excellent electromagnetic wave absorbers, it is necessary to consider their microstructure, various electromagnetic parameters and attenuation properties for the optimization and improvement of their wave absorption performance.

### Impedance Matching

To ensure strong absorption of electromagnetic waves, the impedance matching is a critical parameter that can be achieved. It necessitates that the input impedance of absorbers (Z_in_) closely approximates the impedance of free space (Z_0_), typically expressed as Z_in_/Z_0_ = 1, according to the impedance matching characteristic (Z = Z_in_/Z_0_) equation:

3 $$ \left| {Z_{{{\text{in}}}} /Z_{0} } \right| = \left| {\sqrt {\frac{{\mu_{{\text{r}}} }}{{\varepsilon_{{\text{r}}} }}} \tanh \left[ {\frac{2\pi jfd}{c}\sqrt {\mu_{{\text{r}}} \varepsilon_{{\text{r}}} } } \right]} \right| $$

When |Z_in_/Z_0_| is close to 1, it signifies a perfect impedance matching condition, allowing incident EMWs to efficiently enter the absorber. This concept is vital for designing effective EMW absorbers, because only those that satisfy impedance matching conditions could have maximal EMW penetration and subsequent absorption [6].

Another criterion for evaluating the impedance matching of absorber is the delta function (|Δ|), where the values of K and M are determined by complex permittivity (ε_r_) and complex permeability (μ_r_). When |Δ| approaches zero, it indicates excellent impedance matching. Achieving this match relies on regulating absorbers' electromagnetic properties and striking a delicate balance between dielectric and magnetic properties. Although the widely used |Z_in_/Z_0_| criterion is convenient and universal, it can be ineffective when Z_in_ and Z_0_ values are significantly disparate, even if |Z_in_/Z_0_| equals one. In contrast, the delta function offers a more complex yet robust measure of impedance matching. Importantly, |the approximate values for Z_in_ and Z_0_ are used for the consideration of |Δ|, ensuring that the criterion remains effective and avoids losing its efficacy [22].

4 $$ |{\Delta }| = \left| {\sinh^{2} (Kfd) - M} \right| $$

5 $$ K = \frac{{4\pi \sqrt {\mu^{\prime}\varepsilon^{\prime}} \sin \frac{{\delta_{e} + \delta_{m} }}{2}}}{{c\cos \delta_{e} \cos \delta_{m} }}, $$

6 $$ M = \frac{{4\mu^{\prime}\cos \delta_{e} \varepsilon^{\prime}\cos \delta_{m} }}{{\left( {\mu^{\prime}\cos \delta_{e} - \varepsilon^{\prime}\cos \delta_{m} } \right)^{2} + \left[ {\tan \left( {\frac{{\delta_{m} }}{2} - \frac{{\delta_{e} }}{2}} \right)} \right]^{2} \left( {\mu^{\prime}\cos \delta_{e} + \varepsilon^{\prime}\cos \delta_{m} } \right)^{2} }} $$

### Dielectric Loss

When EMWs interact with dielectric materials, two distinct electron responses come into play, i.e., charge transfer and dipolar polarization, which correspond to conduction and polarization losses, respectively. Charge transfer depends on electrical conductivity, leading to the conversion of EMW energy into heat through micro-currents and resulting in conductance loss. In contrast, dipolar polarization, often associated with crystal defects and interfaces, undergoes polarization relaxation when exposed to EMWs, which significantly attenuates EMWs and contributes to polarization loss [23].

The dielectric loss is related to ε_r_ in Eqs. (7) and (8) with parameters σ, τ, ε_s_, and ε_∞_, where σ, τ, ε_s_, and ε_∞_ are the electric conductivity, relaxation time, static permittivity, and permittivity at an “infinite” high frequency, respectively. The TMDs-based EMW absorbers, characterized by their transition from semiconductive to conductive behaviors, enable efficient charge transport under the influence of electromagnetic fields, leading to the generation of localized currents. On the other hand, TMDs possess vacancies, adatoms, grain boundaries, and impurities, providing numerous dipoles and polarization sites that facilitate the "polarization-relaxation" of charges and consequently lead to the dissipation of EMW energy. Furthermore, the lamellar structure inherent in TMDs facilitates the formation of heterointerfaces and hierarchical structures, thereby enhancing interfacial polarization [24]. Polarization loss and conduction loss are effectively characterized by Cole–Cole formula in ε' vs. ε'' plots. The semicircles serve as indicators of the strength of multiple polarizations, suggesting that the conduction loss increases with increasing slope of the line segment as the tail of plots approaches a straight line.

7 $$ \varepsilon^{\prime} = \varepsilon_{\infty } + \frac{{\varepsilon_{{\text{s}}} - \varepsilon_{\infty } }}{{\omega^{2} \tau^{2} + 1}} $$

8 $$ \varepsilon^{\prime\prime} = \frac{{\varepsilon_{{\text{s}}} - \varepsilon_{\infty } }}{{\omega^{2} \tau^{2} + 1}}\omega \tau + \frac{\sigma }{\omega \varepsilon }_{0} $$

9 $$ \left( {\varepsilon^{\prime} - \frac{{\varepsilon_{{\text{s}}} + \varepsilon_{\infty } }}{2}} \right)^{2} + (\varepsilon^{\prime\prime})^{2} = \left( {\frac{{\varepsilon_{{\text{s}}} - \varepsilon_{\infty } }}{2}} \right)^{2} $$

### Magnetic Loss

Magnetic loss is another crucial parameter that characterizes the electromagnetic absorption materials, indicating that the materials' response to alternating electric fields could result in the irreversible conversion of a portion of incident electromagnetic wave energy into thermal energy. The strength of magnetic loss is typically measured using the magnetic loss tangent (tanδ_μ_ = μ''/μ') and magnetic permeability. Various mechanisms contribute to magnetic loss, including eddy current loss, natural resonance, exchange resonance, hysteresis loss, and domain wall resonance [6].

Firstly, eddy current loss occurs at high-frequency electromagnetic fields, generating internal eddy currents within the material that lead to the dissipation of electromagnetic energy in the form of heat, as described by Lenz's law. Natural resonance predominates in materials such as ferrites, which occurs when the angular frequency determined by the magnetocrystalline anisotropy of the material approaches that of the incident electromagnetic field. Hysteresis loss predominantly depends on the material's magnetic coercive field, where higher coercive fields result in more significant energy conversion and, consequently, greater magnetic loss. Domain wall loss pertains to the resonance frequency of the material's domain wall matching that of the incident electromagnetic field, leading to continuous energy absorption. Hysteresis loss and domain wall resonance are typically insignificant in the 2–18 GHz frequency range and can be disregarded. Eddy current loss, which can be evaluated using the C_0_ parameter, mainly contributes to magnetic loss when the C_0_ value remains relatively unchanged when the frequency changes.

10 $$ C_{0} = \mu^{\prime\prime}\left( {\mu^{\prime}} \right)^{ - 2} f^{ - 1} = 2\pi \sigma \mu_{0} d^{2} $$

Moreover, exchange resonance typically occurs at higher frequencies (10–18 GHz) than natural resonance (2–10 GHz). Therefore, multiple resonances and eddy current loss are the primary sources of magnetic loss in magnetic TMDs-based electromagnetic wave absorbers [25, 26].

## Microstructures and Electrical Properties of VIB- and VB-Group TMDs

The majority of TMDs exhibit a layered structure consisting of alternating layers of sulfur atoms and transition metal atoms. Within this structure, covalent bonds firmly link the transition metal atoms and sulfur atoms, forming the foundational units for bulk TMDs. Meanwhile, these building units are interconnected through vdW forces. It's worth noting that the binding force between these building units is relatively weak, rendering them susceptible to easy separation. Such atomic structure provides the possibility for the preparation of TMD monolayer from its bulk counterpart. The TMD monolayer has showed some structural and electrical properties not possessed by TMD bulks, such as large specific surface area, various crystal phase structures, and tunable band structure, due to the nano-scale confinement effect [27]. Typically, TMDs with distinct crystal structures exhibit varying electronic properties, including differences in their band structures, density of states, and electron spins. Specifically, the band structure plays a crucial role in determining the electrical conductivity of these materials. TMDs characterized by a narrow band gap tend to exhibit high carrier concentration and enhanced electrical conductivity. Moreover, lattice vibrations also affect electrical conductivity since migrating carriers can experience scattering by phonons, resulting in reduced material conductivity. Furthermore, defects are also one of the key structural features affecting the electrical properties of materials, altering the electronic and optoelectronic characteristics of TMDs. For example, in case of material defects, the charge distribution near the defects tends to become uneven. This feature can lead to the formation of dipoles and the occurrence of polarization-relaxation transitions under the influence of an applied electric field, which ultimately attenuates electromagnetic waves [12].

**Fig. 1:**
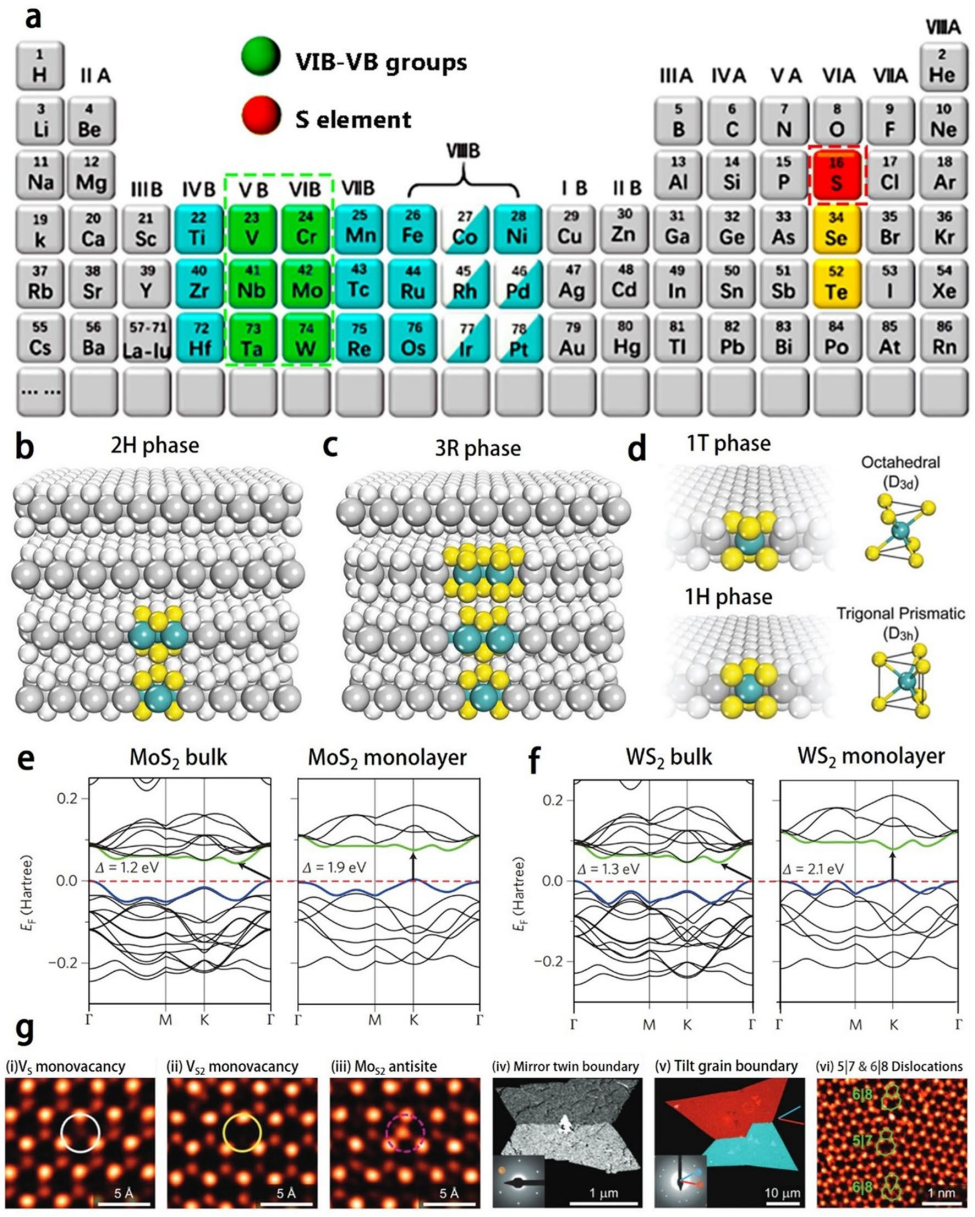
**a** VIB- and VB-group transition metal disulfides highlighted by green and red boxes in the periodic table. The crystal structures of TMDs: **b** hexagonal polytype, 2H-MX_2_, **c** rhombohedral polytype, 3R-MX_2_. **d** Polytypes of monolayer TMDs and their corresponding coordination units, 1T-MX_2_; 1H-MX_2_. Adapted with permission [14], Copyright 2018 The Royal Society of Chemistry. The band structures of **e** MoS_2_ and **f** WS_2_ for bulk and monolayer. Reproduced with permission [47], Copyright 2012 Macmillan publishers Limited. **g** Commonly observed point defects (three on the left) and line defects (three on the right) in TMDs. Reproduced with permission [53, 55], Copyright 2013 American Chemical Society; Copyright 2013 Nature Publishing Group

Recently, the VIB-group and VB-group TMDs (Fig. 1a) have become research hotspots in the field of electromagnetic wave absorption, especially molybdenum sulfide (MoS_2_), tungsten sulfide (WS_2_), vanadium sulfide (VS_2_) and niobium sulfide (NbS_2_). In the following sections, the composition, microstructure, and electrical properties of TMDs are described in detail.

### Electrical Properties of TMDs from Bulk to Monolayer

The crystal structure of bulk TMDs exhibit a variety of polytypes and stacked polytypes (a special case of polytypes), the latter determined by the stacking order of the MX_2_ single layer (encompassing three atomic layers, X-M-X). Bulk TMDs can exist with two primary phases, i.e., the 2H-phase, characterized by hexagonal symmetry, and the 3R-phase, which exhibits rhombohedral symmetry. Both of these phases are semiconducting in nature but differ in their stacking sequences [14], as shown in Fig. 1b-c. Here, we use MoS_2_ as a representative example to elucidate the microstructural and electrical properties of TMDs. For example, bulk MoS_2_ is generally with the 2H phase, which is stacked in the sequence of AbA-BcB, where the uppercase letters represent sulfur atoms and the lowercase letters represent molybdenum atoms. On the contrary, the 3R-phase MoS_2_ is stacked in the sequence of AbA-BcB-CdC. From the X-ray diffraction (XRD) map, it is possible to distinguish between the 2H and 3R crystalline phases, since (h00) plane diffraction with h = 3n ± 1 is only observed in the 2H crystalline phase [28]. At high temperatures (about 1000 °C), the 3R phase transforms into the 2H phase, while the 2H phase remains stable until it reaches the melting point [29].

As shown in Fig. 1d, 2D TMD (TMD monolayer) is with either 1T or 1H phase, which belongs to trigonal or hexagonal symmetry, respectively. The quantum effects induced by the atomic thickness of 2D TMDs enable them to possess unique mechanical, optical, electrical, and magnetic characteristics that are quite different from those of bulk TMDs. Notably, the atoms of single-layer TMDs are of high exposure, which provides great possibilities for tuning their electronic structures, phases, compositions by chemically doping, functionalizing their surfaces, or applying electrostatic potentials [27]. For instance, the MoS_2_ monolayer is mainly with the 1H phase, which exhibits a triangular prismatic symmetry with respect to Mo atoms. When the alkali metal is embedded in the monolayer, the crystal structure of MoS_2_ transforms into the 1T phase, which exhibits an octahedral symmetry with respect to Mo atoms. Such transformation in crystal structure allows 2D MoS_2_ to switch between a semiconductive state (1H phase) and a metallic state (1T phase). There are several techniques to distinguish between the 1H and 1T phases of 2D MoS_2_, such as high-resolution scanning transmission electron microscopy (HR-STEM) [30]. Remarkably, 1T-phase MoS_2_ is in a substable state that converts to 1H-phase MoS_2_ upon aging in air for two months [31] or annealed at 300 °C in argon for one hour [16, 17]. While there are some reports on the stabilization of the 1 T-phase MoS_2_ that it can be stabilized by doping rhodium [33] and vanadium [34]. For 2D WS_2_, it basically has similar polymorphic transition and structural properties to MoS_2_.

**Fig. 2:**
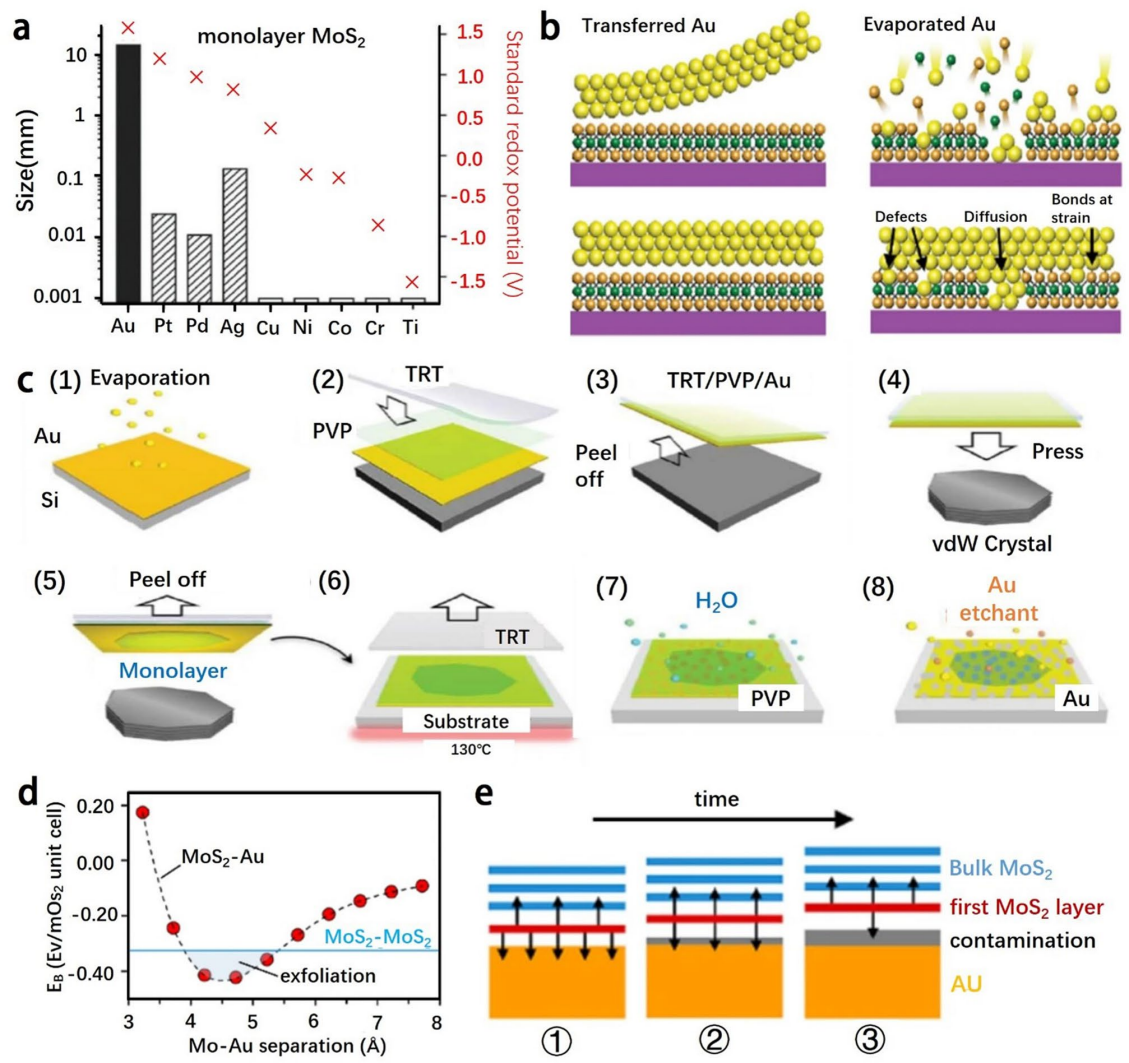
**a** Size of the exfoliated monolayer of MoS_2_ with different metals was compared. Adapted with permission [64], Copyright 2020 Wiley–VCH GmbH. **b** Two different contact principles for Au-MoS_2_ are introduced: direct contact (left) and evaporation contact (right). Adapted with permission [66], Copyright 2018 Macmillan Publishers Limited, part of Springer Nature. **c** (1)-(9) are schematic diagrams of the mechanical exfoliation process of MoS_2_ with gold as the exfoliation substrate. Reproduced with permission [138], Copyright 2020 American Association for the Advancement of Science. **d** Relationship between the Au–S distance and the binding tendency of MoS_2_. **e** Effect of gold surface contamination on the Au–S distance with time. Adapted with permission [63], Copyright 2018 American Chemical Society

Different from those of VIB-group TMDs, the 2H and 1T phases of VB-group TMDs are in the metallic states. Due to their unique electrical properties, VB-group TMDs show great promise as a single-component wave absorber, which eliminates the cumbersome compounding process in favor of the application in the field of wave absorption. NbS_2_ has a layered structure and is a typical member of VB-group TMDs. It has superconductivity and unique electronic states, as well as optical properties and magnetisms [35–40]. Theoretical [41] and experimental [42] studies have shown that 2D NbS_2_ is a desirable material for realizing the dielectric loss of EMW absorption due to its abundant active sites (at its edges and on the substrates) and excellent electrical conductivity (as regulated by its number of layers) [38, 43]. The features of Gibbs free energy, unique charge density wave (CDW) [43–45], and anisotropic structure of 2D NbS_2_ [46] facilitate the realization of its unique EMW absorption properties. VS_2_ is another VB-Group TMDs which is superior to VIB-group WS_2_ and MoS_2_ with respect to electronic transport capacities and unusual electronic states. VS_2_ possesses better microwave absorption performance due to its electron mobility and abundant active sites [6].

Figure 1e-f shows the energy band structures by first principles calculations for bulk and single-layer 2H-phase MoS_2_ and WS_2_. Bulk MoS_2_ and WS_2_ are indirect bandgap semiconductors, which has a bandgap of 1.2 and 1.3 eV, respectively. As the layers of MoS_2_ or WS_2_ is reduced to obtain atomic thicknesses, its bandgap is enhanced and changed from indirect to direct bandgaps [47]. The bandgap of MoS_2_ and WS_2_ monolayer is 1.9 and 2.1 eV, respectively. Based on density-functional theory (DFT), the results of first-principles calculations can be used to explain the transition of MX_2_ from a smaller indirect bandgap to a larger direct bandgap, and have been frequently used to unravel more details of the electron-band structure of MX_2_ and MX_2_ monolayer materials. Represented by MoS_2_ as a compound of the VIB group, bulk MoS_2_ is an indirect bandgap semiconductor with a valence band maximum (VBM) located at the Γ-point and a conduction band minimum (CBM) located almost midway between the Γ-point and the K-point, as shown in Fig. 1e [48]. By reducing the number of layers in MoS_2_, the bandgap becomes larger but remains between Γ and K points until the layers have an atomic thickness. For the MoS_2_ monolayer, both its VBM and CBM are located at the K point, resulting in a direct bandgap [48].

### Electrical Properties of TMDs with Defect

The crystalline TMDs nanomaterials have many outstanding physicochemical properties, which could be altered by structural defects as introduced during the process of crystal growth or in the subsequent post-processing steps. If the defects are well adjusted or controlled, the TMDs can be tuned to have new functionalities that are useful in practical applications. Figure 1g shows the point and line defects commonly observed in various TMDs.

For MoS_2_, due to the fact that the atomic number of molybdenum (Z_m_ = 42) is larger than that of sulfur (Z_s_ = 16), the bright areas in the annular dark-field scanning transmission electron microscopy (ADF-STEM) images are attributed to molybdenum atoms, while dim areas are attributed to sulfur atoms. As shown in Fig. 1g(i), the removal of a single S atom from the MoS_2_ top layer leads to the appearance of a dimmer sulfur spot at the location of the spots ascribed to S atoms, indicating the formation of a single sulfur vacancy defect (V_S_). If another S atom is subsequently removed from the bottom layer, the corresponding S site is not occupied, which is manifested as the disappearance of the sulfur spot, indicating the formation of disulfide vacancy (V_S2_), as shown in Fig. 1g(ii). In case an exotic atom such as molybdenum atom substitutes for a disulfide vacancy and develops an antisite defect, then a new bright dot appears, as shown in Fig. 1g(iii). According to theoretical predictions, covering sulfur vacancies in MoS_2_ with Nitrogen (N), Phosphorus (P), Arsenic (As), and Antimony (Sb) could cause N-doping, while covering sulfur vacancies with Fluorine (F), Chlorine (Cl), Bromine (Br), and Iodine (I) could cause P-doping [49]. It has been reported that there are significant improvements in the electrical properties and photoluminescence of oxidized molybdenum disulfides [50–52], resulting from the defects introduced into MoS_2_.

Grain boundary, as a planar defect, is another important defect in VIB-group TMDs, which could have significant influences on physical and chemical properties of TMDs. Generally, grain boundaries are categorized according to the inclination angle (θ) between two neighboring grains. Under the extreme condition of θ = 60°, alternative name the inclination angle at which a twin grain boundary is formed (Fig. 1g(iv)) [53], there are two types of dislocations at the grain boundary, namely 4|4 and 4|8 dislocations. TMDs with 4|4 dislocations could exhibit metal properties, while those with 4|8 dislocations show antiferromagnetic semiconducting properties [27, 28]. The mobility of electrons at the inclined grain boundary is consistently reduced (Fig. 1g(v)) [53, 56]. Noteworthy, in the presence of sulfur- or molybdenum-rich atoms, low-angle tilted (5|7) grain boundaries also may include 6|8 or 4|6 dislocations and be nonmagnetic. For example, Fig. 1g(vi) shows an 18.5° tilt grain boundary containing 5|7 and 6|8 dislocation, which grow from localized atomic structures rich in sulfur atoms [55].

In summary, the transition of TMDs from bulk materials to monolayers brings about several notable effects, which include a substantial increase in specific surface area, resulting in the exposure of a greater number of functional groups. Such increased exposure facilitates the elongation of electromagnetic wave propagation paths within the material, leading to enhanced electromagnetic losses. Additionally, the phase structure undergoes a transformation from the original semiconductor phases (2H and 3R) to 1T metallic phase and 1H semiconductor phase, imparting greater flexibility in conductivity modulation. Furthermore, this structural transition often entails an enlargement of the band gap, shifting it from indirect to direct band gaps. Imperfections within the internal microstructure of TMDs can give rise to dipole moments, which, upon interaction with external electromagnetic waves, induce polarization losses, effectively attenuating the electromagnetic waves. In general, because of its unique diversity of chemical and physical properties, and the potential for these properties to be fine-tuned through synthesis, VIB-group and VB-group TMDs has become a viable platform for the investigation of numerous research topics about EMW absorption.

## Various Synthesis Routes for TMDs-Based Nanomaterials

2D TMDs have potential applications in high-speed electronic circuits, large-scale integrated optoelectronic devices, and catalysis. However, the fabrication and research of 2D materials still require further development for applications in various fields, such as the large-scale controllable fabrication of high-quality single-layer or few-layer TMDs and the improvement on their transmission properties compared to commercial materials. Viet Phuong Pham et al. [57] reported the application of doped MoS_2_ structures in photodetectors, transistors, thin-film photovoltaic devices, p-n junctions, non-volatile multi-bit data memories, ultra-sensitive sensors, and photocatalysts. Based on this work, it is particularly important to improve the synthesis methods of commonly used TMDs nanomaterials [58].

Successful preparation of high quality nanomaterials based on TMDs is crucial for their application in EMW absorption. Several preparing methods have been developed in the past decade to implement morphologically and structurally controlled synthesis of TMDs nanomaterials. Moreover, the incorporation of carbon materials, metal oxides, metal sulfides, and other materials with TMDs nanomaterials also facilitates their potential value in the area of EMAs because of the synergy effect of the diverse materials [9, 59, 60]. In this section, we summarize some commonly used methodologies for synthesizing TMDs nanomaterials presented in Table 1, including the top-down approaches through exfoliating bulk TMDs and the bottom-up approaches such as hydro/solvothermal methods and vapor-phase deposition synthesis routes.

**Table 1:** Comparative summary of different preparation methods and routes of TMDs in terms of reaction conditions and characteristics

Techniques/Materials	Characteristics	Solvent	Critical conditions	Environment	Precursors	Methods	Refs
**Top-down**							
Au assisted-exfoliation	High quality but low yield limit/suitable for fundamental researches or fabrications		Exfoliating force > interlayer forceCLQB: beyond 0.5 eVMoS_2_: ca.-0.34 eV	20 − 23 °C/50 − 70% humidity	Bulk TMDs	Mechanical exfoliation	[46–51]
Li assisted-chemical exfoliation	Time consuming(6-48 h)/low yield with impurities	Hexane	Li + intercalation	60 °C /argon-filled glove box	Bulk TMDs/organolithium compounds e.g.: BuLi, MeLi, LiBH_4_	Chemical exfoliation	[53]–[57]
Li assisted- electrochemical exfoliation	Complicated (assembly of battery cells)/high yield with impurities	LiPF_6_ as electrolyte/EC, DMC, NMP as solution	Li + intercalation conducted in lithium battery test system	Room temperature/Ar-filled glove box	Bulk TMDs/Li foil		[58–60]
LAAL	Short of time(1 h), high yield with plentiful vacancies and edges	Liquid nitrogen	Li + ion intercalation assisted by ammonia gas	5 × 10^–4^ Pa/argon-filled glove box	Bulk TMDs/ lithium pieces		[61, 62]
**Down-top**							
Monolayer or few-layers NbS_2_	Products with high-quality, low-defect, and layer controllable nanostructures			~ 650 °C, SiO_2_/Si substrate with h-BN	NbCl_5_/S	CVD	[63–67]
MoS_2_/WS_2_			Temperature control switched growth between vertical and lateral	Lateral: 650 °C Vertical: 850°CSiO_2_/Si as substrate	Te + W, MoO_3_/S		
MoS_2_/h-BN				h-BN: 1000°CNi-Ga/Mo foil as substrateMoS_2_: 80°Ch-BN films on Ni-Ga as substrate	h-BN: NH_3_-BH_3_MoS_2_: Mo foil/H_2_S		
WS_2_/rGO	Low cost and facile, products with easy-controlled structure	Water		210 ℃	WCl_6_, CH_3_CSNH_2_/ GO dispersion	hydrothermal	[43–45]
WS_2_/NiO					WCl_6_, CH_3_CSNH_2_/ NiO dispersion		[68–71]

### Exfoliation from the Bulk TMDs

TMDs nanosheets (NSs) are readily available by exfoliation from the bulk TMDs since the vdW interactions between neighboring layers are weak. Exfoliating bulk TMDs enables the production of laminar TMDs nanosheets with a generous surface area and a wealth of active sites.

A direct method for obtaining single or few layer structures for TMDs is to exfoliate the bulk TMDs through a physical method, i.e., mechanical exfoliation. With highly polarizable electrons, the density dispersion interactions in TMDs are enhanced and are termed covalent-like-quasi-bonding (CLQB) [61]. In comparison with vdW interactions in TMDs, CLQB interactions have more directionality, i.e., that involves wavefunction hybridization [61]. The interaction energy can achieve more than 0.5 eV per unit cell [62] and is thus expected to overcome interlayer vdW interactions in laminar materials (e.g., MoS_2_ has an interfacial binding energy of about -0.34 eV) [63]). Some noble metals have highly polarization properties, and some studies have also shown that gold has a better exfoliating effect on MoS_2_ than other precious metals [64] (Fig. 2a). Therefore, Johnston et al. proposed to evaporate gold directly onto MoS_2_ [65], so that gold and MoS_2_ can be better bonded. Also, Max Heyl et al. used gold atoms as an example to construct a model for ordinary binding and evaporation binding, and found that [66] damage, defects or new chemical bonds in MoS_2_ may also be introduced when hot gold atoms bombarded the MoS_2_ interface (Fig. 2b). Velický et al. used gold as a substrate for mechanically exfoliating MoS_2_ [63]. The Au–S distance in the resulting materials (3.5 Å) was detected to be markedly greater than that of typical covalent Au–S bond (~ 2.2 Å), confirming that the enhanced vdW interaction under the action of Au was still smaller than the covalent binding energy. They also obtained the range of Au–S distance required to overcome the interlayer binding energy of MoS_2_ on the basis of the results of their experiments (Fig. 2d). It turns out that the gold surfaces could build up contaminations between the Au–S layers due to the pollution of thiols and airborne carbon; and the exfoliation process should be limited to a certain period of time (< 6 min in air), otherwise the distance between the Au–S layers was increased (Fig. 2e) [63]. Nonetheless, the mechanical exfoliation has some obvious shortcomings. Although high-quality 2D TMDs NS can be obtained by conventional mechanical exfoliation, the process is also limited in terms of yield due to the limitation of the quality of the starting TMD crystals. In addition, scotch tape exfoliating on some substrates such as silicon wafers does not provide sufficient interaction forces.

**Fig. 3:**
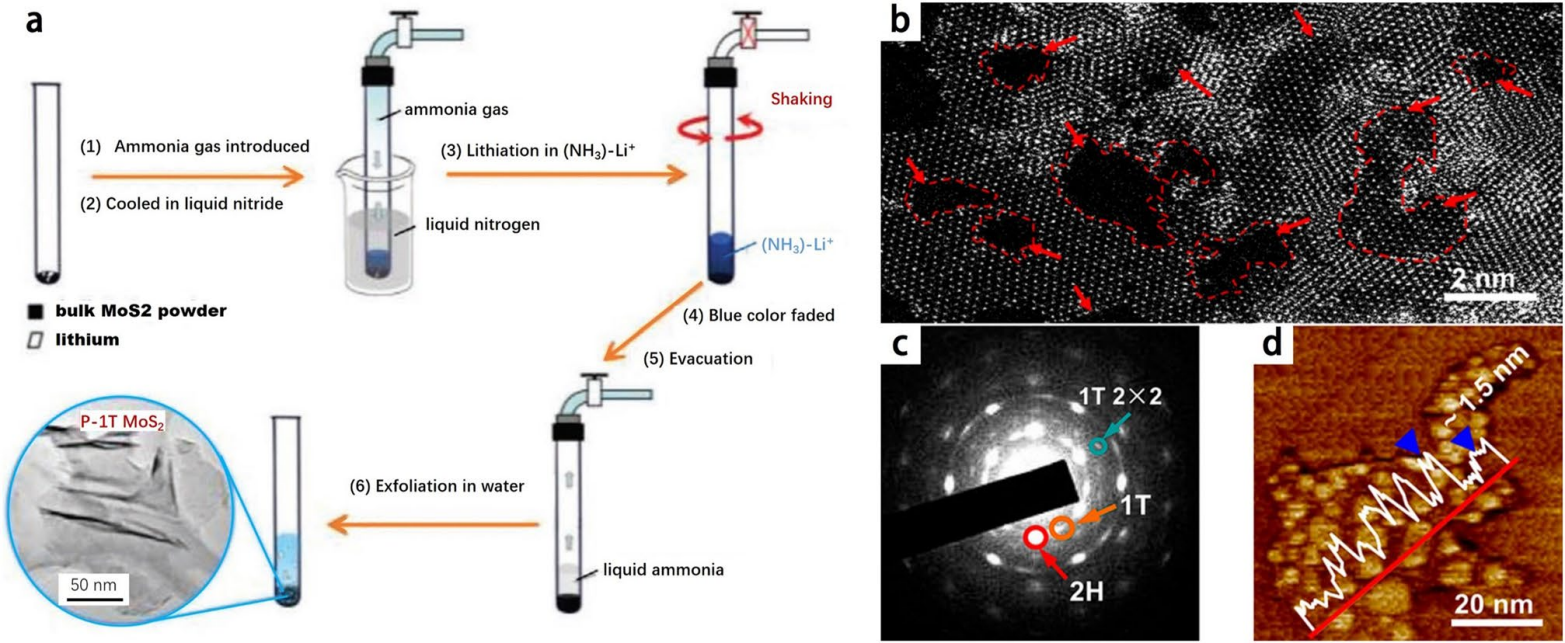
**a** A diagrammatic drawing of LAAL processes. **b** Corresponding high-resolution STEM image **c** SAED patterns and **d** atomic force microscopy (AFM)image of exfoliated MoS_2_ NSs. Reproduced with permission [76], Copyright 2016 American Chemical Society

In order to solve the problems mentioned above, Li^+^ ion intercalation-assisted stripping has also been extensively employed and is considered an efficacious technique for obtaining laminated TMDs materials for massive scale. Representative Li^+^ ion intercalation-assisted stripping processes consist of three procedures: To achieve the desired outcome, the following steps are followed: i) incorporation of Li^+^ ions into the interlayer region of the bulk TMDs; ii) submersion of the compounds containing Li^+^ ions in a solvent; iii) application of ultrasonication to the compounds [67]. On the basis of the above processes, multiple synthesis routes have been explored, including chemically lithium-ion intercalation-assisted stripping of organolithium compounds such as butyllithium (BuLi) [68–71], methyllithium (MeLi) [72], or lithium borohydride (LiBH_4_) [70], electrochemical Li^+^ ion intercalation-assisted exfoliation conducted within a lithium battery test cell [73–75]. It should be kept in mind that the Li^+^ ion intercalation-assisted exfoliation method does have a drawback. It can leave behind traces of foreign ions in the TMDs nanosheets, making their complete removal a challenging task. Song et al. introduced an innovative approach called the liquid ammonia assisted lithiation (LAAL) technique, which proved to be highly effective in achieving the exfoliation of bulk MoS_2_ powders and obtaining ultrathin 2D MoS_2_ nanosheets [76, 77] (Fig. 3a). By combining lithium metal and MoS_2_ in a quartz tube under argon protection and cooling it with liquid nitrogen, they initiated a reaction that changed the color of the mixture. After removing the ammonia gas and introducing water, they successfully obtained ultrathin 2D MoS_2_ nanosheets (Fig. 3b-d). LAAL technique offers several advantages: 1) The reaction process is visibly indicated by a distinct color change within a short time (usually within 1 h), eliminating the need for additional indicators. 2) The method yields a high percentage (approximately 82%) of ultrathin TMDs nanosheets. 3) The resulting nanosheets exhibit abundant sulfur vacancies and increased edges, which enhance their electromagnetic application performance.

In summary, 2D TMDs used as electromagnetic wave absorbers can be synthesized by exfoliating bulk TMDs. Various synthesis routes have been proven to be effective, such as mechanical exfoliation, Li^+^ ion intercalation-assisted exfoliation, LAAL technology, especially the lithiation process. However, a lot of efforts have to be devoted to design and control the morphology and layer number of TMDs NSs.

### Chemical Vapor Deposition

Chemical vapor deposition (CVD) is a process where gaseous or vaporized reactants, along with necessary gases, are introduced into a reaction chamber. A chemical reaction takes place on the substrate's surface, resulting in the formation of a thin film. The following examples contain both TMDs and their heterostructures with this approach.

**Fig. 4:**
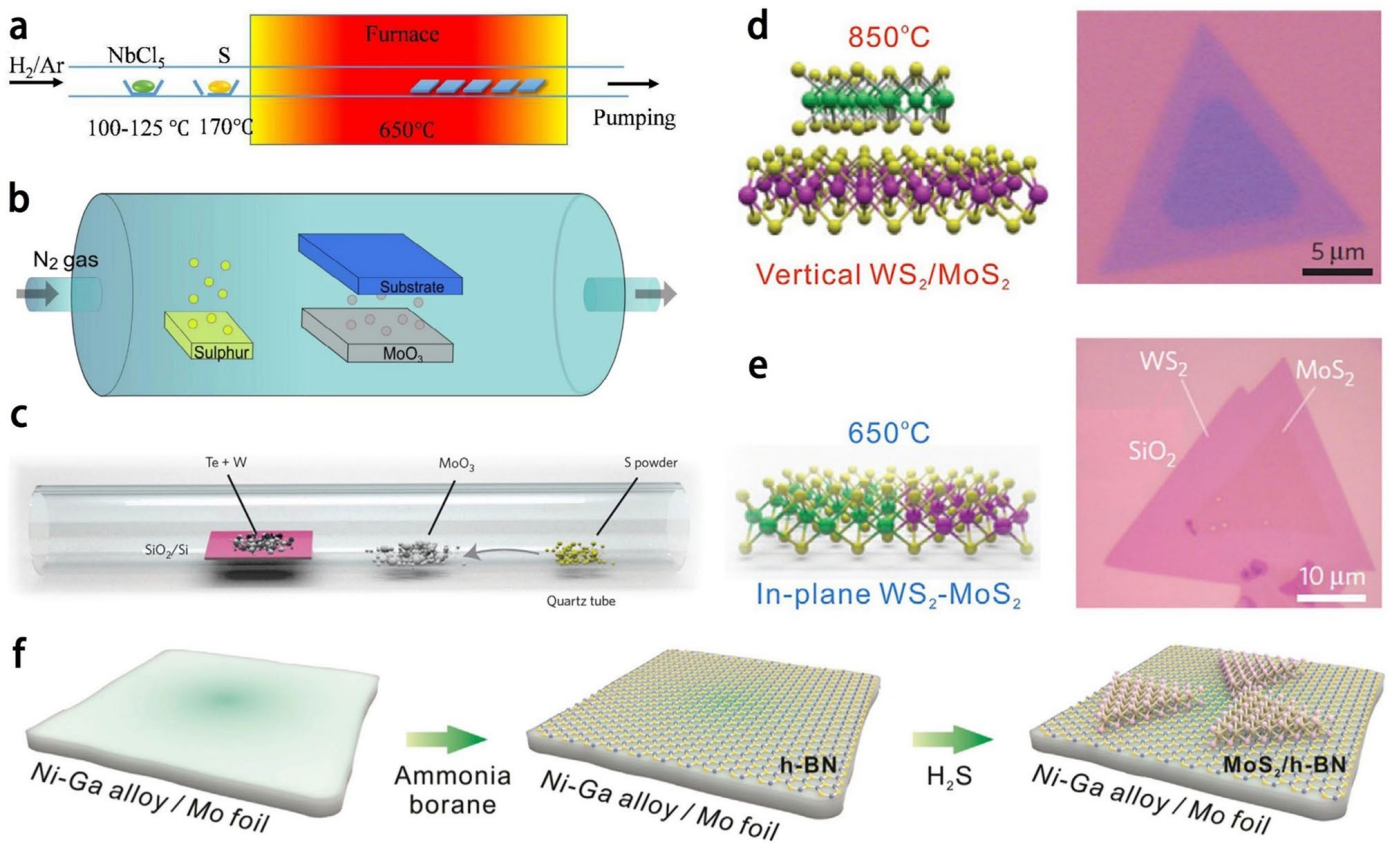
**a** Illustration of CVD system for NbS_2_ synthesis. Adapted with permission [36], Copyright 2017 The Royal Society of Chemistry. **b** A typical powder-source CVD setup for producing 2D TMDs nanosheets. **c** Schematic diagram showing the CVD growth process. **d** Schematic of vertical WS_2_/ MoS_2_ heterostructure synthesized at 850 °C and its typical optical image. **e** Schematic of in-plane WS_2_-MoS_2_ heterostructure grown at 650 °C and its typical optical image. Adapted with permission [78], Copyright 2014 Macmillan Publishers Limited. **f** Schematic showing the CVD growth of MoS_2_/h-BN heterostructures on Ni–Ga. Adapted with permission [81], Copyright 2016 American Chemical Society

As shown in Fig. 4a, NbS_2_ was synthesized by CVD, by using NbCl_5_ (with heating temperatures 100–125 °C) and S (with heating temperatures ~ 170 °C) as precursors. Monolayer and few-layer NbS_2_ can be obtained. It is worth noting that the precursor should be moved to a quartz tube as soon as possible (within 30 s) after weighted since NbCl_5_ is very sensitive to moisture. The center of the furnace can be heated to maintain a temperature of ~ 650 °C. The SiO_2_/Si substrate with hexagonal boron nitride (h-BN) flakes is placed downstream inside the furnace at a deposition temperature of 650–500 °C [36].

A typical CVD configuration, as illustrated in Fig. 4b, employs N_2_ as the carrier gas. The vaporization of the powder source results in the formation of gaseous precursors that are transported by the carrier gas to the substrate. Subsequently, the precursors undergo a chemical reaction, leading to the growth of 2D TMDs on the substrate. However, in the context of powder-sourced CVD growth, the reaction of the precursors before reaching the targeted sublayer is inevitable. This poses a significant challenge in achieving uniformity and homogeneity of the 2D TMDs over a large area on the substrate. In addition, direct CVD growth is more advantageous because large single-crystal 2D sheets with higher crystalline quality could be prepared. The growth process of 2D TMDs using CVD can be summarized in four key steps: (1) Evaporation: The chemical source undergoes evaporation and is introduced into the carrier gas. (2) Reaction: The chemical species reacts with sulfur, resulting in the formation of MO_2-x_S_x_ (0 ≤ x ≤ 1) compounds within the carrier gas, where M represents the transition metal. (3) Diffusion: The MO_2-x_S_x_ (0 ≤ x ≤ 1) compounds diffuse within the carrier gas and reach the target substrate. (4) Migration and Nucleation: The MO_2-x_S_x_ (0 ≤ x ≤ 1) compounds migrate and react with the substrate surface, initiating the nucleation and growth of 2D TMD flakes. The quality and uniformity of TMDs growth in CVD are influenced by several critical parameters, including the concentrations of individual chemical species and their mass ratio, the flow rate of the carrier gas, the growth temperature, and the configuration of the source-substrate setup. These parameters play a crucial role in determining the overall characteristics and performance of the resulting TMDs. However, it is still a challenge to control and optimize these factors to tune the nucleation density and sizes of TMDs 2D materials.

In addition, it is possible to realize the switching growth of vertical and lateral TMDs heterostructures by controlling the temperature during the CVD process. Gong et al. [78] successfully achieved the controlled growth of vertical and planar heterostructures between MoS_2_ and WS_2_. They employed W-Te mixed powders, instead of the commonly used WO_3_, as the growth precursor. This innovative approach proved crucial in preventing the formation of Mo_x_W_1–x_S_2_ alloys. In their work, Fig. 4c demonstrates the manipulative growth process, while Fig. 4d illustrates a schematic diagram of a vertically stacked heterostructure consisting of a WS_2_/MoS_2_ bilayer, along with its distinctive optical pattern. The bilayer region (dark purple) is clearly distinguishable from the MoS_2_ monolayer (light purple). Furthermore, Fig. 4e showcases the schematic and morphology of an in-plane WS_2_-MoS_2_ heterostructure, highlighting the distinct transverse interface between the single-layer MoS_2_ and WS_2_. The temperature-dependent growth strategy can be summarized as follows: Initially, the nucleation and growth of WS_2_ exhibit lower rates compared to MoS_2_ due to the lower W vapor pressure and limited solubility of W in liquid Te within the temperature range of 650 to 850 °C. Consequently, a monolayer of MoS_2_ is initially formed on the substrate. Subsequently, the growth behavior of WS_2_ is highly influenced by the reaction temperature, leading to two distinct growth scenarios: (1) At lower temperatures (650 °C), WS_2_ nucleation and growth are challenging and slow. However, the strong chemical bonding between WS_2_ and the MoS_2_ edge results in a significantly reduced nucleation energy compared to that on the MoS_2_ surface. As an consequence, an in-plane heterostructure is formed. (2) At higher temperatures (850 °C), the nucleation barrier can be overcome by the increased thermal energy. Under these conditions, thermodynamically more stable products are favored. The preference shifts towards the formation of vertical WS_2_/MoS_2_ heterostructures due to the substantial vdW energy generated by the stacking. Thus, by precisely controlling the growth temperature, selective formation of either in-plane WS_2_-MoS_2_ heterostructures or vertical WS_2_/MoS_2_ bilayers can be achieved.

Dielectric heterostructures could also be constructed by the CVD approach, such as TMDs/h-BN [79, 80]. The utilization of commonly used h-BN growth substrates, such as Ni or Cu, in the synthesis of TMDs can pose challenges due to the formation of sulfides during subsequent sulfur-rich TMD synthesis. The intense sulfur-metal bonding can lead to the decomposition of pre-grown h-BN membranes. To address this limitation, Fu et al. [81] presented a pioneering approach by utilizing sulfide-resistant metals for the CVD growth of TMDs/h-BN heterostructures. The fabrication process of MoS_2_/h-BN heterostructures, as depicted in Fig. 4f, involved the formation of a Ni-Ga alloy on a Mo foil through thermal annealing. Subsequently, multiple layers of h-BN films were grown on the Ni-Ga substrate. The introduction of H_2_S into the CVD system enabled the growth of MoS_2_ on the h-BN surface. This synthetic route allowed for the direct synthesis of MoS_2_/h-BN heterostructures via CVD without the need for any additional transfer steps.

### Hydro/Solvothermal Method

**Fig. 5:**
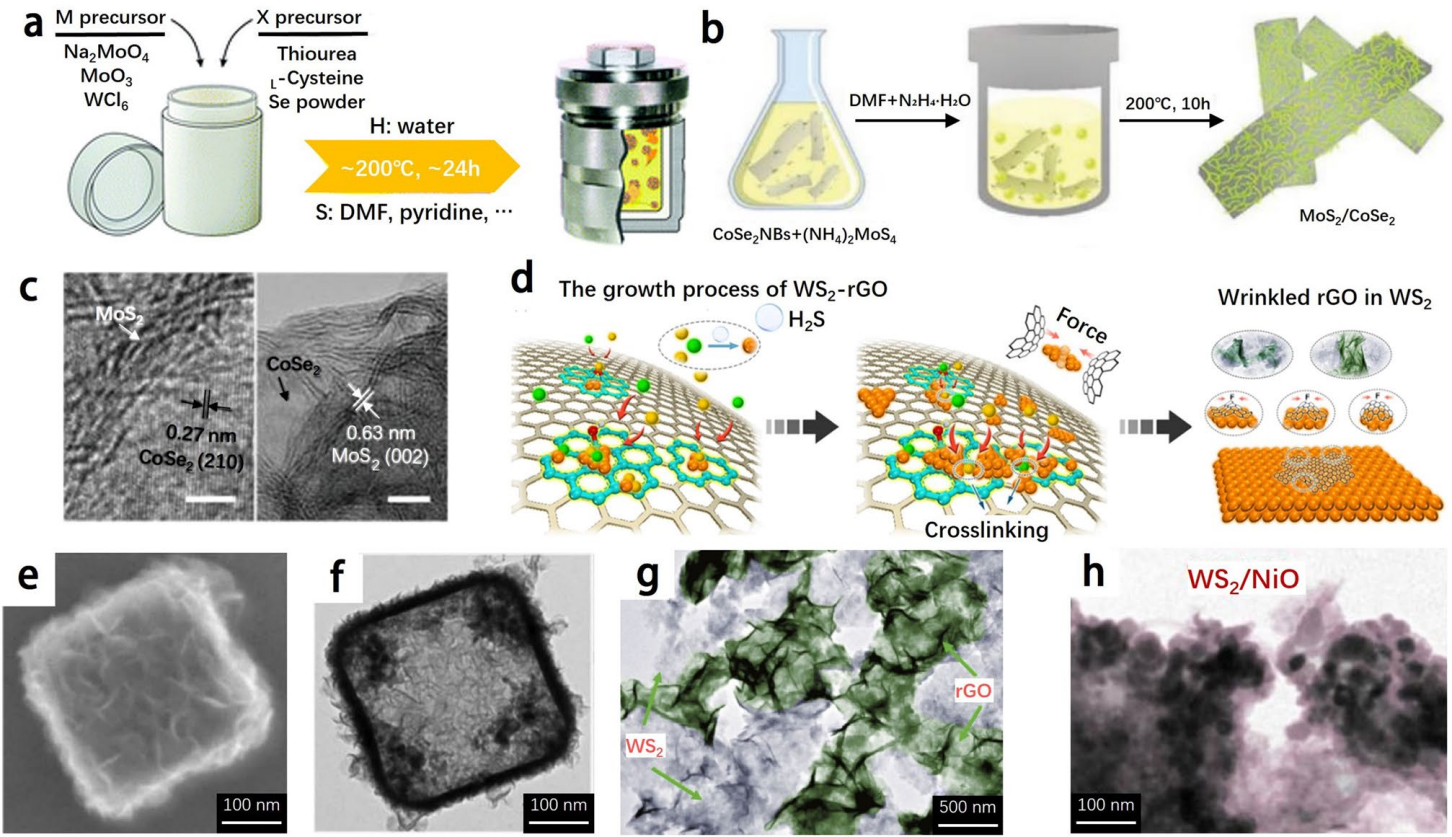
**a** Schematic of the processes during the hydrothermal and solvothermal synthesis routes. Adapted with permission [14], Copyright 2018 The Royal Society of Chemistry. **b** Synthesis route of two-component MoS_2_/CoSe_2_ hybrid catalyst. **c** HRTEM result of the MoS_2_/CoSe_2_ hybrid. Adapted with permission [82], Copyright 2015 Macmillan Publishers Limited. **d** Schematic illustration of the growth process of WS_2_ − rGO architecture which exhibits the process of turning a guest into a host. Adapted with permission [10], Copyright 2019 American Chemical Society. **e** FESEM image and **f** TEM image of the as-synthesized products. Adapted with permission [60], Copyright 2020 WILEY–VCH Verlag GmbH & Co. KGaA Weinheim. **g** TEM images of WS_2_–rGO. Adapted with permission [9], Copyright 2019 Springer. **h** TEM images of WS_2_/NiO [59]. Adapted with permission, Copyright 2019 Elsevier B.V. and Science China Press

The hydro/solvothermal method is a low-cost technique that can be easily operated, and it is one of the facile methods for the large-scale synthesis of TMDs-based nanomaterials. By adjusting experimental parameters such as temperature, reaction time, solvent, metal precursor type, surfactant and other additives, various samples with different morphologies, phases or crystallinities can be obtained. The solvent can be water or an organic solvent, and its boiling point is usually lower than the reaction temperature. As the temperature increases, the reactivity of the solvent increases, and the pressure in the closed system increases, thereby promoting the reaction and increasing the crystallinity. The reaction is therefore usually carried out in a sealed high-pressure vessel, or an autoclave [14] (Fig. 5a).

Hydrothermal reaction is a common strategy for preparing nanostructured materials, and its advantage is the selectivity to the final products. The abundant combinations of reactants will facilitate the construction of hetero-structured nanomaterials, such as MoS_2_/CoSe_2_, which possess abundant interfaces [82] (Fig. 5b-c). The disadvantage of solvothermal reaction is that the product is easily oxidized during the production process, which will affect the purity of the produced material. In order to avoid oxidation, solvothermal reaction is used to synthesize TMDs-based nanomaterials [83, 84]. Due to the relatively low preparation temperature of the hydrothermal reaction, the crystallinity of the prepared material is usually not as good as that prepared at a high temperature such as a solid-state reaction, while the product could occupy more active sites. In addition, due to the closed system, it is difficult to observe the growth phase in the hydro/solvothermal process, and it is a challenge to monitor the reaction process for experimental modification. The hydro/solvothermal synthesis is also very sensitive to experimental conditions, and precise control is required in each reaction stage.

The solvothermal method can be applied to the synthesis of TMDs with different morphologies, which can also be exploited to develop controllable morphologies of TMDs. Metal organic frameworks (MOFs) are used as precursors for the preparation of TMDs. Lou and co-workers [60] synthesized MoS_2_ hollow nano-boxes combined with nickel and cobalt, as shown in Fig. 5e-f, which had very fine morphology and structure.

Certain TMDs nanomaterials synthesized using the aforementioned methods exhibit remarkable EMA properties. In Fig. 5d, the growth process and mechanisms of WS_2_-reduced graphene oxide (WS_2_-rGO) nanosheets are depicted, which are obtained through the "transforming an object into a subject" growth approach [10]. Cheng et al. [9] successfully synthesized 2D WS_2_-rGO heterostructure nanosheets (Fig. 5g) through a facile hydrothermal method. They further investigated the dielectric and EMA properties of these nanosheets. The EMA absorbers, composed of TMDs nanomaterials and wax, demonstrated an optimal reflection loss of -41.5 dB and the effective absorption bandwidth spanning 13.62 GHz (4.38–18 GHz) when the absorber thickness was 2.7 mm. These lightweight WS_2_-rGO nanosheets exhibit promising potential for practical EMW absorption applications. In another study, Zhang et al. [59] developed WS_2_/NiO heterostructured hybrids as EMA materials using a hydrothermal method (Fig. 5h). The hybrid with 20% NiO loading achieved an optimal RL of -53.31 dB at the thickness of 4.30 mm. The enhanced EMA absorption performance of WS_2_/NiO hybrids can be attributed to the addition of magnetic NiO, which results in synergistic magnetic and dielectric losses in the interfacial hybridization between WS_2_ and NiO.

## Structure Engineering Modulation of TMDs

2D TMDs are extremely sensitive to synthesis and processing. The disorder caused by intrinsic defects and the external environment is significant for their industrialization. The intrinsic defects include vacancy defects, antisite defects, and substitution defects, etc., which can be addressed by improving the preparation process. The sources that induce disorder in materials could include the construction of structure, strain, surface roughness, charged impurities, and other factors, which can be adjusted through tuning substrate and protective devices. Therefore, in order to realize the industrial application of 2D TMDs, it is necessary to first develop synthesis and fabrication techniques that can produce large-scale, stable, repeatable, and scalable less-disorder materials under structural control at nanoscales [85].

Among them, structure engineering modulation is a promising field of research that has the potential to revolutionize the way that the materials are designed and developed. By manipulating the material’s structure at nanoscales, we can achieve unprecedented control over its properties, allowing us to create the material with tailored and optimized properties for specific applications. This approach has shown great promise in a wide range of fields, including electronics, photonics, energy storage, and catalysis. Structure engineering modulation can easily and effectively realize the large-scale tuning of materials properties by manipulating their structure at nanoscales, opening-up new possibilities for the development of advanced materials suitable for a wide range of applications.

One of the key advantages of structure engineering modulation is its ability to precisely control the electronic structure of materials. By tuning the thickness, shape, lattice structure, and chemical composition of 2D materials, we can achieve precise control over their band structure, Fermi surface shape, carrier density, and other key electronic properties. Such control is critical to developing high-performance electronics capable of operating at the limits of what is physically possible.

### Construction of Diverse Morphologies and Architectures

In the field of materials science, the development of unique microstructures is highly significant. Researchers have focused on various microstructural designs such as core–shell structures, flower-like spheres, hollow particles, foamed structures, and hierarchical architectures. The deliberate design of these structures enables the formation of interfaces between different materials, polarization at the interfaces, and multiple reflections and scattering. These features are crucial for creating EMW absorbers.

For the EMW absorption of VIB- and VB-group TMDs, the morphology of TMDs plays a significant role in their EMW absorption performance. TMDs with unique structures, including multiple layers, hollow spheres, flower-like formations, and core-sheath configurations, exhibit enhanced electromagnetic wave absorption properties. These peculiar structures enable extended transmission paths for electromagnetic waves within the materials, resulting in increased wave-matter interactions and consequently excellent absorption performance. Moreover, TMDs with anisotropic structures, such as nanowires or nanotubes, often exhibit better absorption properties than isotropic ones such as spherical nanoparticles. The anisotropic materials have larger surface areas and more surface defects, which can enhance the interaction between the microwave radiation and the materials. The thickness and size of TMDs also significantly influence their microwave absorption properties. In general, it is possible to control the shape, thickness, and size of TMDs to optimize their morphology for high-performance EMW absorption.

**Fig. 6:**
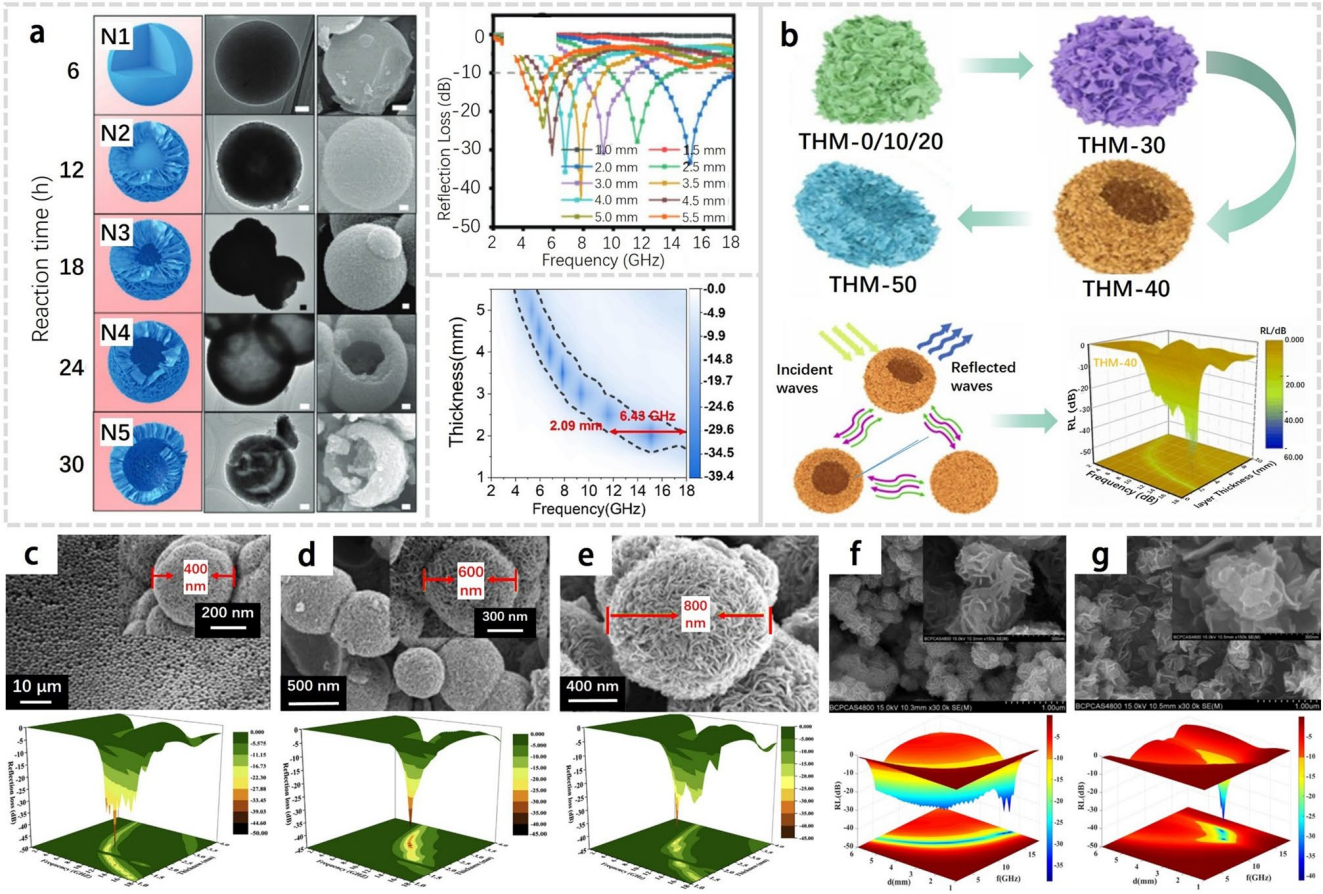
**a** Schematic of the growth mechanisms, reflection loss curves, and corresponding 2D maps of hollow-sphere NbS_2_. Adapted with permission [16], Copyright 2021 Wiley–VCH GmbH. **b** Schematic illustration for the evolution processes of 1T/2H-MoS_2_ nanocomposites affected by adjusting the solvent contents, and their microwave absorption mechanism and performance. Reproduced with permission [15], Copyright 2022 Elsevier B.V. **c-e** SEM images and 3D surface plots of reflection loss for H-MoS_2_, HH-MoS_2_ and MoS_2_/C. Reproduced with permission [86], Copyright 2018 Elsevier B.V. **f-g** SEM image and three-dimensional representation of electromagnetic wave absorption for flower-shaped and flake-shaped MoS_2_ nanoparticles. Reproduced with permission [87], Copyright 2019 Elsevier B.V

Specifically, Cheng et al. [16] synthesized NbS_2_ with a hierarchical hollow-sphere structure which showed good EMW absorption capability with an RL_min_ value of 43.85 dB and an EAB of 6.48 GHz, as shown in Fig. 6a. Liang et al. [15] successfully synthesized hollow flower-like 1T/2H MoS_2_ microspheres through a simple hydrothermal process. These microspheres exhibited exceptional microwave absorption properties, characterized by a minimum reflection loss value of -56.32 dB at 17.16 GHz, with a matching thickness of 1.85 mm. The remarkable microwave absorption performance and effective absorption bandwidth at smaller matched thicknesses can be attributed to the unique flower-like hollow nanostructures, high dielectric loss, and favorable impedance matching properties of the material. Qi et al. [86] prepared MoS_2_ with different microstructures, which possess different absorbing properties. Among them, MoS_2_/C microspheres showed the best absorbing performance with a minimum reflection loss value of 44.67 dB at a thickness of 1.4 mm and an EAB up to 3.32 GHz, as shown in Fig. 6c-e. Yang et al. [87] synthesized flower-shaped MoS_2_ nanoparticles, which displayed an EAB of 7.6 GHz and a minimum RL value of -39.20 dB at 17.60 GHz, as shown in Fig. 6f-g.

**Fig. 7:**
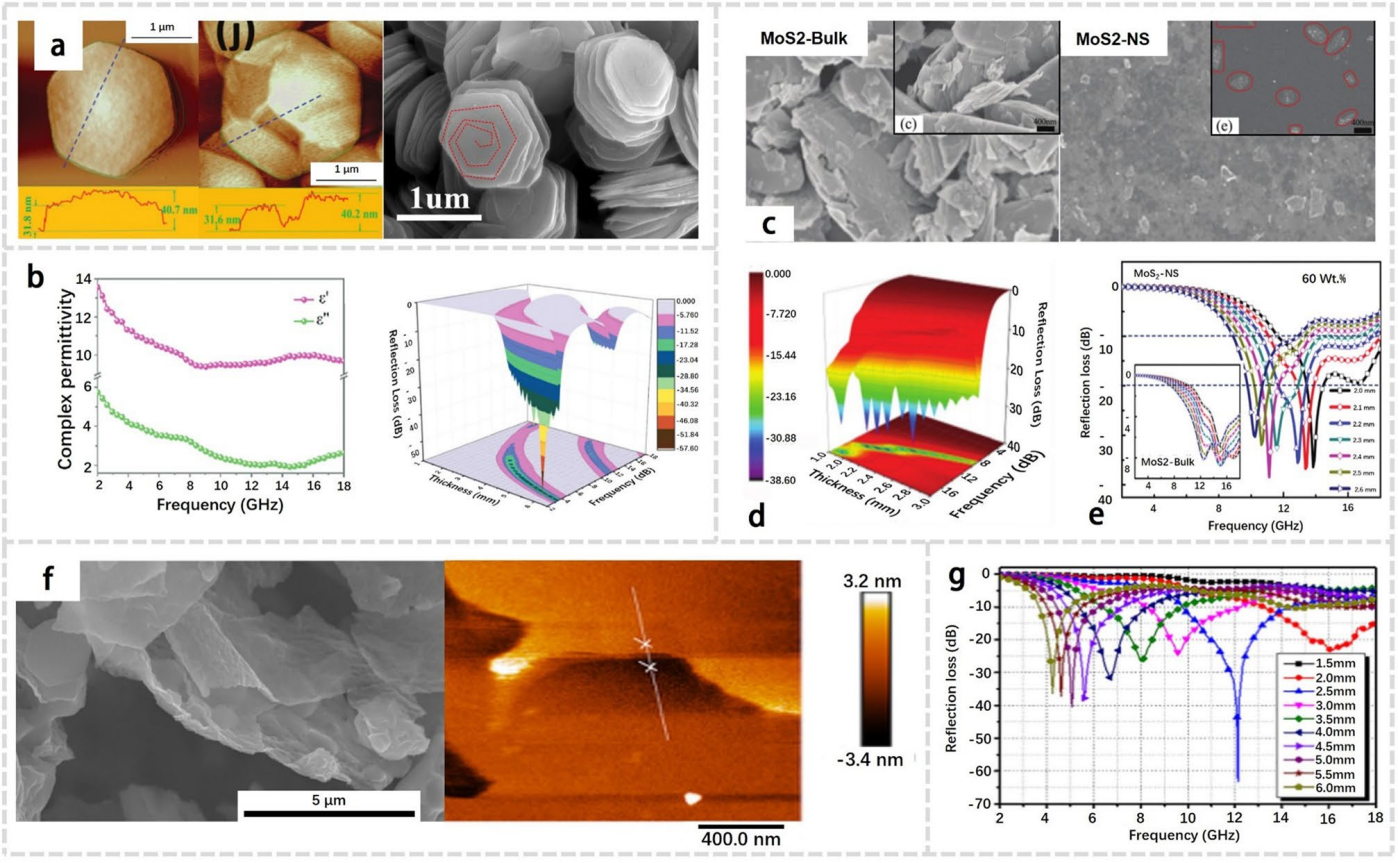
**a** AFM images and SEM images of VS_2_. **b** Complex permittivity and calculated frequency-dependent RL values in 3D plots of VS_2_. Adapted with permission [8], Copyright 2020 The Royal Society of Chemistry. **c** SEM images of MoS_2_-Bulk and MoS_2_-NS. **d** 3D plot of the RL values versus the frequency and thickness of MoS_2_-NS/wax composites with 60 wt% loading. **e** RL of MoS_2_-Bulk/wax and MoS_2_-NS/ wax composites with 60 wt% loadings at different thicknesses. Adapted with permission [89], Copyright 2015 The Royal Society of Chemistry. **f** SEM image and AFM image of FL-WS_2_. **g** RL properties of FL-WS_2_ with a content of 30% in the composites. Adapted with permission [88], Copyright 2018 AIP Publishing

Because of their larger surface areas per unit volume which enhanced microwave radiation-materials interaction, thinner materials generally exhibit higher microwave absorption efficiency than thicker ones. Recently, Zhang et al.[8] produced coaxial stacking VS_2_ nanosheets through hydrothermal synthesis route, as shown in Fig. 7a. They obtained nanosheets with different thicknesses and crystal structures by controlling the reaction conditions, thereby modulating the electromagnetic parameters and achieving excellent performance in C- and Ku-band dual-frequency absorption, as shown in Fig. 7b [1]. Similarly, Wu et al. [88] synthesized WS_2_ nanosheets with a thickness of 3–4 µm, and the composite consisting of 30 wt% few-layer (FL) WS_2_ exhibited an effective electromagnetic absorption bandwidth of 4.6 GHz with the thickness of 2.5 mm, with an RL_min_ value up to -63.0 dB, as shown in Fig. 7f-g. The results demonstrate the importance in controlling the thickness of TMDs for the optimization on their EMW absorption properties.

Smaller particles generally exhibit higher microwave absorption efficiency than larger ones, due to their higher surface area to volume ratio which increases the likelihood of interaction between the microwave radiation and the materials. Moreover, smaller particles often occupy more surface defects, which can further enhance their absorption properties. Recently, Ning et al. [89] employed the top-down exfoliation method to produce few-layer MoS_2_ nanosheets (MoS_2_-NS) from bulk MoS_2_ (MoS_2_-Bulk). They discovered that a composite of MoS_2_-NS and wax, with a loading of 60 wt%, exhibited an impressive minimum reflection loss of -38.42 dB at a thickness of 2.4 nm. This value was nearly four times higher than that of MoS_2_-Bulk/wax composites. Furthermore, the corresponding EAB of the MoS_2_-NS/wax composite reached up to 4.1 GHz (9.6–13.76 GHz), as evidenced in Fig. 7c-e. These findings highlight the importance of particle-size control for optimizing the EMW absorption performance of TMDs.

To conclude, the microwave absorption performance of TMDs nanomaterials from VB group to VIB group can be improved by controlling their thickness, size, and shape. With further research and development on the microstructures, TMDs nanomaterials may have even more promising prospects in the fields of EMW absorption.

### Adjustment on Phase Structures

**Fig. 8:**
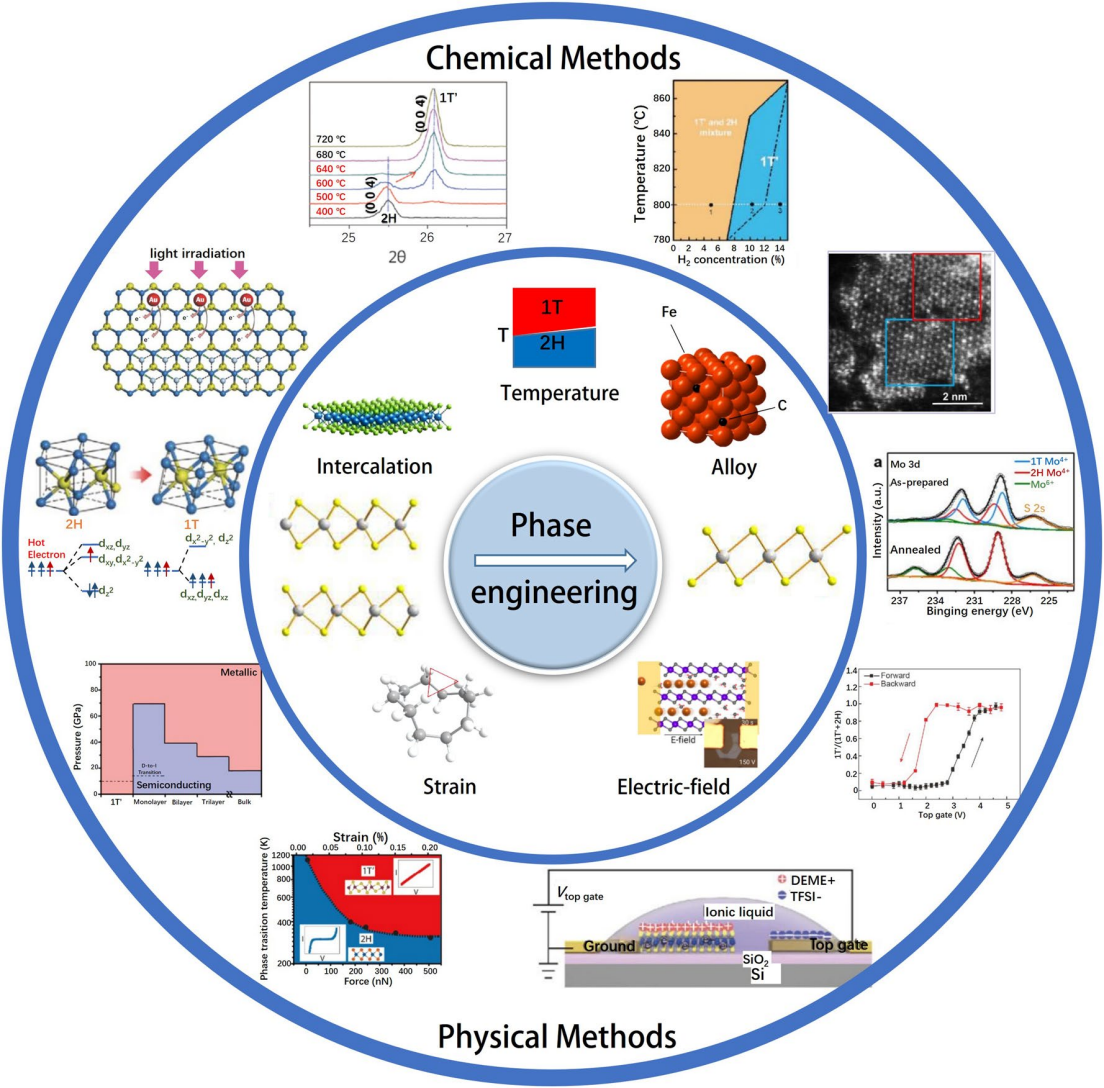
Overview of phase engineering approaches for 2D TMDs

2D TMDs exhibit distinct chemical and physical properties, such as high catalytic activity, semiconductor behavior, superconductivity, magnetic ordering, and ferroelectric polarization, owing to their polycrystalline nature. These properties vary depending on the specific phases of the TMDs. For instance, 2H-MoS_2_ is an n-type semiconductor, while 1T-MoS_2_ is a metal. Bulk crystals of 1T-MoS_2_ also demonstrate superconductivity at 4 K, whereas 2H-MoS_2_ is diamagnetic and 1T-MoS_2_ is paramagnetic. Consequently, comprehending the phase transition properties becomes crucial for exploring the physicochemical characteristics of TMDs. Phase engineering techniques for TMDs encompass chemical methods involving ion insertion and alloying, as well as physical approaches such as strain application and electric field modulation (Fig. 8). However, ion insertion methods have predominantly been employed for phase tuning of TMDs in the context of microwave absorption. This review primarily focuses on the phase engineering of TMDs with 1T and 2H phases, highlighting their potential in tailoring the electromagnetic wave absorption properties of microwave absorbing materials, with particular emphasis on ion intercalation and its influence on the microwave absorption applications of TMDs.

**Fig. 9:**
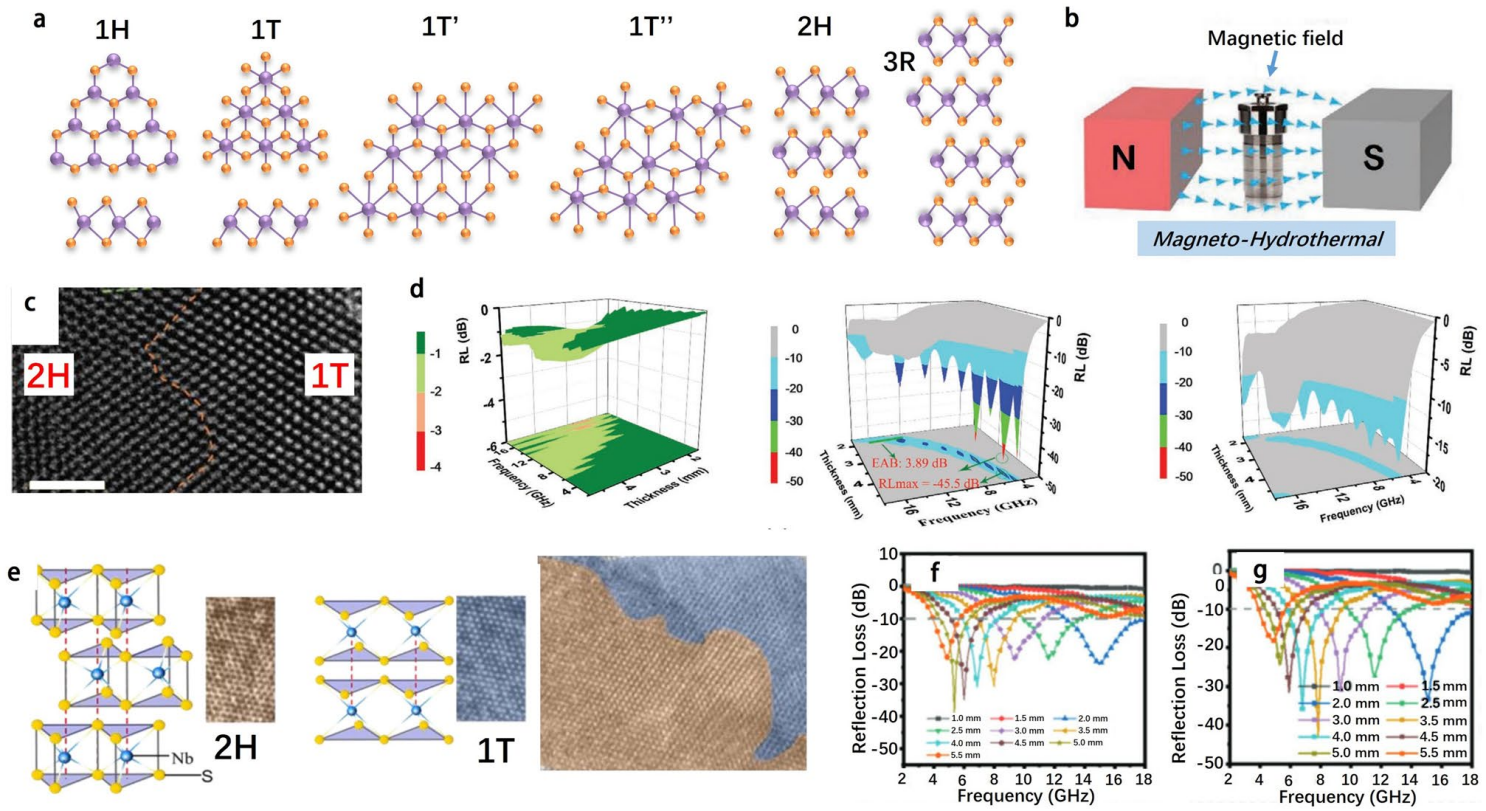
**a** Structures and phases of 2D TMDs. Adapted with permission [90], Copyright 2019 Science China Press and Springer-Verlag GmbH Germany, part of Springer Nature. **b** Schematic illustration of the synthesis of MoS_2_ with pure or mixed phases. **c** STEM images of 1T-50% absorbers. **d** 3D RLs-f curves and 2D projection plots of 2H-100%, 1T-50%, and 1T-100% absorbers with 50 wt% loadings at thicknesses of 1.6–4.8 mm. Adapted with permission [19], Copyright 2021 Wiley–VCH GmbH. **e** Schematic diagram of crystal structures and HRTEM image of 1T phase and 2H phase in NbS_2_. **f-g** Reflection loss curves of samples with different phase ratios. Adapted with permission [16], Copyright 2021 Wiley–VCH GmbH

2D TMDs can take on different phase structures depending on the oxidation state of the transition metals. Generally, 2D TMDs have two basic phases: the 1H phase with D_3h_ symmetry, which is a trigonal phase, and the 1T phase with D_3d_ symmetry, which is an octahedral phase. Moreover, the 1T phase of TMDs can undergo structural distortions, resulting in 1T' and 1T" phases. The stacking and alignment of 1H phase layers could lead to the formation of 2H and 3R phases (Fig. 9a). Different filling configurations of d orbitals of transition metals give rise to different atomic structures of TMDs and different stabilities of 2D TMDs [90]. Thus, 2D TMDs materials with different phases could have different thermodynamic stabilities.

MoS_2_ is a promising material for EMW absorption due to its defect induced polarization that could be caused by vacancies and high specific surface area. However, bare MoS_2_ still suffers from low conductivity and limited active sites. Ning et al. [19] developed a simple method called magneto-hydrothermal process to prepare MoS_2_ with different 2H/1T mass ratios, which introduced abundant phase interfaces and optimized the conductivity, as shown in Fig. 9b. Figure 9c shows the scanning transmission electron microscopy (STEM) image of the 1T-50% absorber, clearly demonstrating the presence of both 1T and 2H phases within a single MoS_2_ sheet. MoS_2_ with 50% 2H and 50% 1T phases (intercalated) shows the best EMW absorption performance with an RL_min_ value of -45.5 dB, which is ten times of that of pure 2H phase, as shown in Fig. 9d. The optimized EMW absorption performance is related to strong charge transfer at the 2H/1T phase interfaces and moderately optimized conduction losses. This work demonstrates the effectiveness of phase engineering of MoS_2_ in EMW attenuation.

NbS_2_ is a type of vdW bonded transition metal dichalcogenides with excellent electrical conductivity and abundant active sites, making it an ideal material for electromagnetic wave absorption. Zhang et al. developed a new solvothermal method for preparing NbS_2_ hollow nanospheres containing vertically stacked nanosheets. They had a mixed multiphase structure, with both 2H and 1T phases coexisted, which could improve their EMW absorption performance. High resolution transmission electron microscope (HRTEM) images are used to identify the 2H and 1T crystalline phases of NbS_2_, which are represented in the brown and blue areas, respectively, as shown in Fig. 9e [16]. The 1T to 2H phase ratios of samples shown in Fig. 9f, g are 1.37:1, 1.08:1, respectively. It is found that the closer the ratio of 1T phase to 2H phase is to 1:1, the better the EMW absorption performance.

**Fig. 10:**
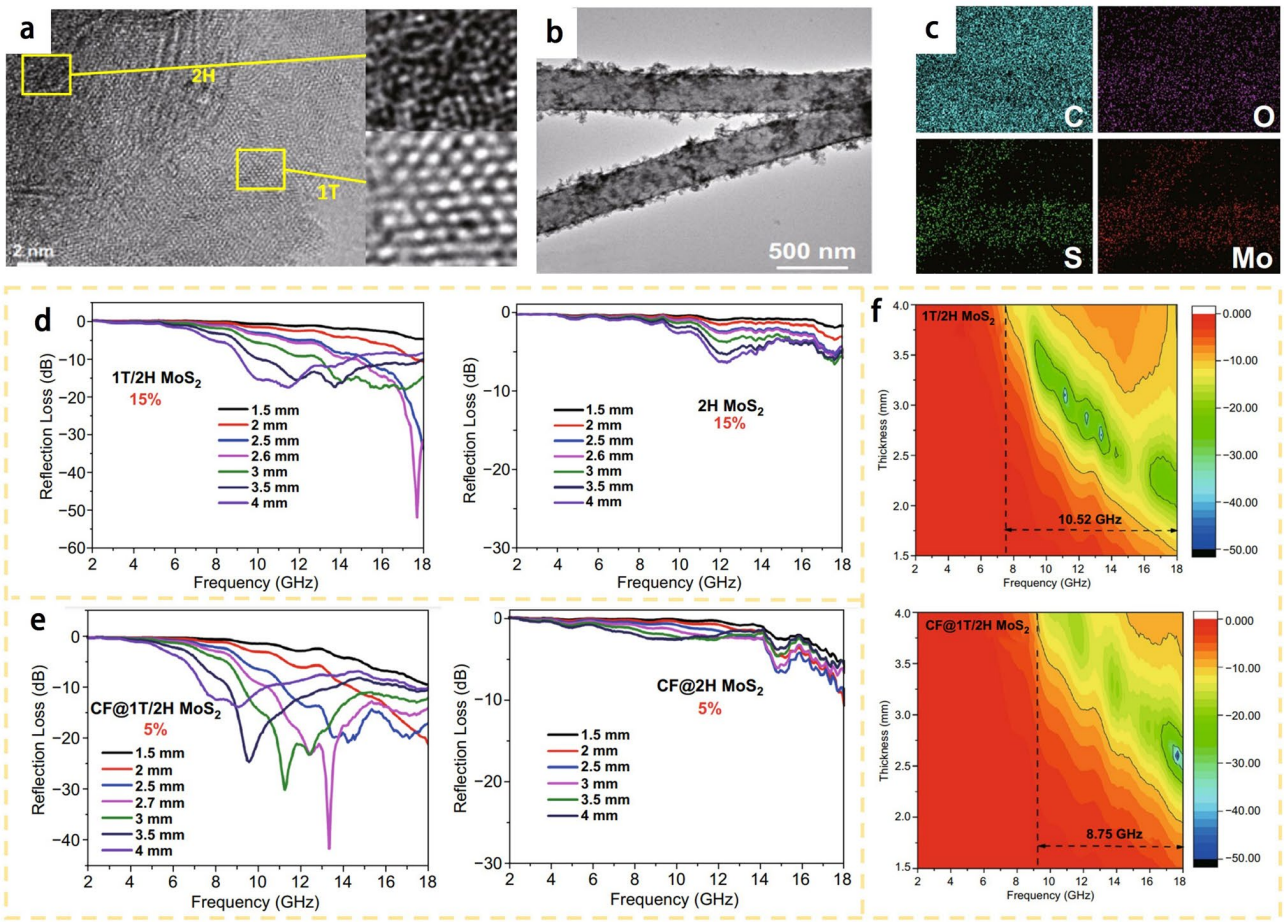
**a** HRTEM images of 1T/2H MoS_2_, and enlarged images of the selected yellow area. **b** TEM images of CF@1T/2H MoS_2_. **c** element mapping of C, O, S, Mo. **d** Calculated refection loss of 1T/2H MoS_2_ and 2H MoS_2_ with the matrix loading of 15wt%. **e** CF@1T/2H MoS_2_ and CF@2H MoS_2_ with the matrix loading of 5wt%. **f** Corresponding contour maps of 1T/2H MoS_2_ (15%) and CF@1T/2H MoS_2_ (5%). Reproduced with permission [18], Copyright 2021 Springer

Yan et al. [18] developed a simple hydrothermal method to synthesize 1T/2H MoS_2_, where the 1T phase was induced through intercalation of guest molecules and ions. This process is illustrated in Fig. 10a. After the in-situ growth of carbon fiber (CF)@1T/2H MoS_2_, the flakes coated on the carbon fiber surface can be clearly seen, as shown in Fig. 10b. Figure 10c shows that the elements are evenly distributed. Ultra-thin 1T/2H MoS_2_ were grown on carbon fibers using a similar approach, resulting in CF@1T/2H MoS_2_ and CF@2H MoS_2_. Figure 10d demonstrates the significant differences between 2H MoS_2_ and 1T/2H MoS_2_ based absorbers with a filling amount of 15%. The minimum reflection loss value of 1T/2H MoS_2_ can reach as low as -52.7 dB at 17.7 GHz when the thickness is 2.6 mm, while 2H MoS_2_ with 15% filling content shows almost no electromagnetic wave absorption. Similarly, Fig. 10e shows the reflection loss of CF@1T/2H MoS_2_ and CF@2H MoS_2_ based absorbers with 5% filling content, revealing that CF@1T/2H MoS_2_ has an extremely high absorption performance with an RL_min_ value of -43 dB at 13.4 GHz and with only 5% filler loading in the absorbers. In contrast, CF@2H MoS_2_ based absorbers with 5% filling content shows negligible electromagnetic wave absorption. The electromagnetic wave absorption capabilities of the two phases are compared clearly in Fig. 10f. When the thickness of 1T/2H MoS2 (15%) based absorbers is 1.5 to 4 mm, the corresponding EAB can reach 10.52 GHz, which is equivalent to that in the X- (8–12 GHz) and Ku- (12–18 GHz) bands. When CF is added, the EAB range becomes 9.25 to 18 GHz.

**Fig. 11:**
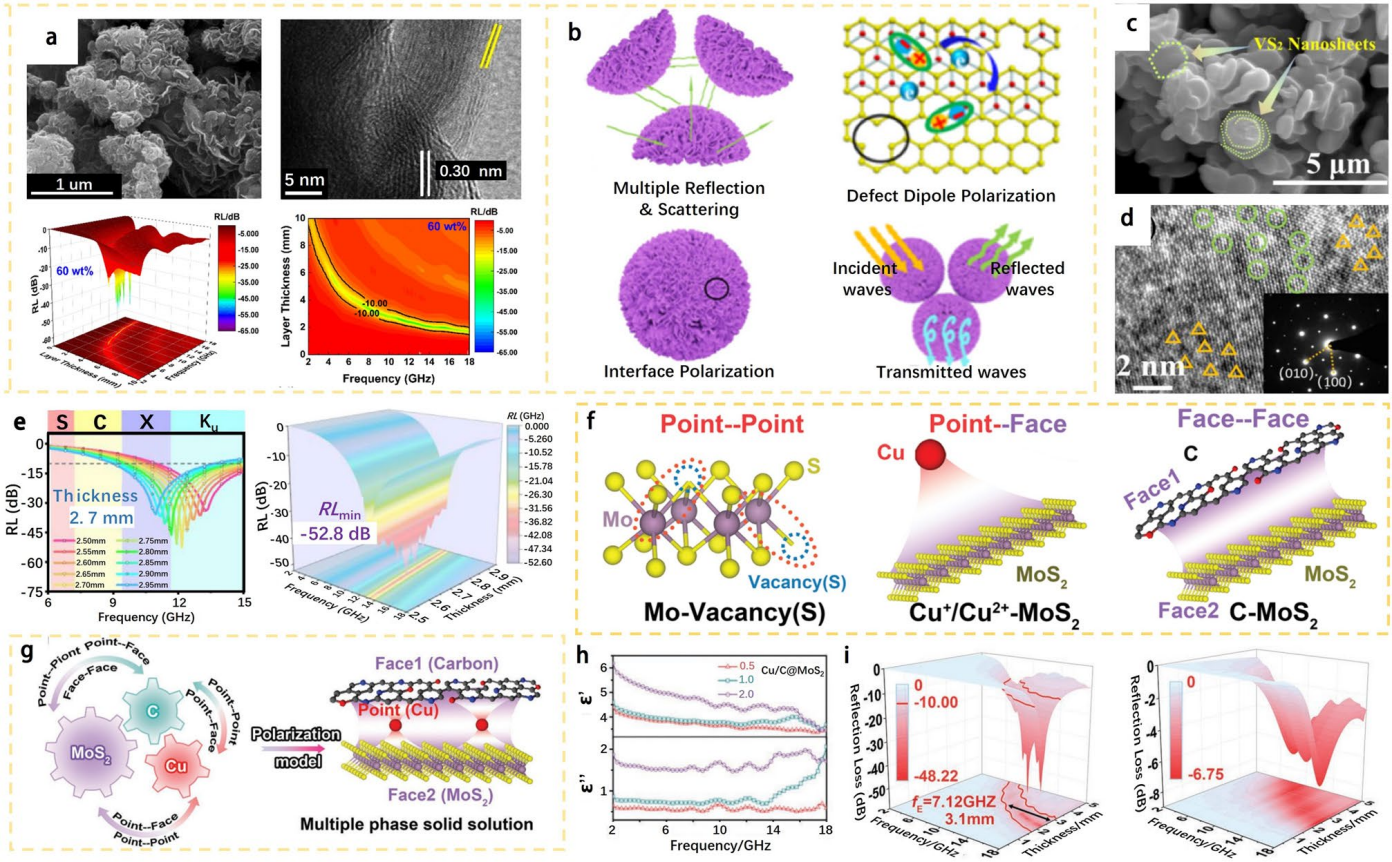
**a** FESEM, HRTEM images and electromagnetic wave absorption performance of the 1T/2H-MoS_2_ nanostructures. **b** Mechanism of the 3D flower-like 1T/2H-MoS_2_ nanostructures. Reproduced with permission [17], Copyright 2021 American Chemical Society. **c** SEM images of VS_2_/GDC hybrids. **d** HR-TEM images of VS_2_ nanosheets (The inset is a SADE image). **e** RL and contour map for the corresponding 3D RL plots of VS_2_/15GDC700°C with a thickness of 2.50–2.95 mm. Adapted with permission [91], Copyright 2023 Elsevier B.V. on behalf of The Chinese Ceramic Society. **f** Schematic structures of polarization centers in MoS_2_, Cu@MoS_2_, and C@MoS_2_, respectively. **g** Schematic representation of polarization models in the multiple-phase MoS_2_ solid solution. **h** ε′ and ε″ values of Cu/C@MoS_2_. **i** 3D RL values of Cu/C@MoS_2_ 2.0 and Cu/C@MoS_2_ 0.5, respectively. Adapted with permission [92], Copyright 2022 Wiley–VCH GmbH

Wang et al. [17] developed a flower-like nanostructure of 1T/2H-MoS_2_ and investigated its potential applications for microwave absorption. The field emission scanning electron microscopy (FESEM) and HRTEM image reveals a flower-like nanostructure of the material, consisting of both 2H phase (with lattice fringes measuring 0.27 nm) and 1T phase (with lattice fringes measuring 0.30 nm) of MoS_2_. Notably, the 1T phase component of the material demonstrates a high ability for electron transfer and dielectric loss, facilitating effective attenuation of electromagnetic waves (Fig. 11a). Furthermore, depicted in Fig. 11b, the flower-like nanostructure of the 1T/2H-MoS_2_ material, along with its substantial specific surface area, facilitates enhanced reflection and scattering of incident waves through multiple interactions. This results in an increased number of contact points for electromagnetic waves. Simultaneously, the flower-like nanostructure of the 1T/2H-MoS_2_ material exhibits abundant interface polarization, while the presence of defect-dipole polarization proves highly advantageous for the dissipation of electromagnetic waves.

Wang et al. [91] introduced a novel approach utilizing glucose as a carbon precursor to selectively coat the surface of stacked VS_2_ nanosheets with glucose-derived carbon (GDC). This process involved a hydrothermal treatment followed by high-temperature carbonization, as depicted in Fig. 11c. The resulting VS_2_ nanosheets exhibited a combination of both 1T (depicted as green circles) and 2H phases (depicted as yellow triangles), as shown in Fig. 11d. Notably, the presence of a substantial quantity of 1T phases significantly enhanced the material's conductivity and optimized impedance matching, thereby enabling effective EMW absorption. These findings are illustrated in Fig. 11e.

### Design and Regulation of Defects

Surface defects significantly affect the chemical and physical attributes of 2D TMDs, which are widely presented in various 2D TMDs. They may be formed during the growth of samples, or are created during post-processing. The defects increase the number of dipoles and afford multiple polarization relaxation centers for electromagnetic wave attenuation, which results in effective improvement of dielectric loss, promotion and enhancement of conduction loss, dipole polarization, interface polarization, multiple reflections, and scattering, which further promotes the effective absorption of electromagnetic waves.

Gao et al. [92] proposed a new metal–organic synergistic interaction method, using 2H-MoS_2_, polydopamine (PDA), and Cu as raw materials to prepare a series of materials that could simultaneously control S vacancies, Cu interstitials, N substitutions, and carbon/MoS_2_ heterointerfaces. The nanostructured Cu/C@MoS_2_ solid solution could have the cooperative polarization loss of various points and planes/sites, which are elaborated as follows: First, S vacancies induce typical defect polarization in MoS_2_; Second, the difference in dielectric properties between carbon matrix and MoS_2_ film causes interface polarization; Third, the electron cloud structure and its relative spatial position on the interface between Cu ions and MoS_2_ will also undergo regular deformation under the action of an external electromagnetic field, thereby realizing the corresponding polarization phenomenon (Fig. 11f). Furthermore, the polarization loss model for Cu/C@MoS_2_ (Fig. 11g) was established, which described the point-point, point-plane and plane-plane heterogeneous sites constructed by S vacancies in Cu/C@MoS_2_, and the induced cooperative polarization among hetero-sites, interstitial Cu, substituted N, and the heterointerface between carbon and MoS_2_. The model could explain the excellent dielectric loss that the carrier (cavity) activity and interaction with hybridization of Cu and carbon are enhanced. In addition, the dielectric polarization of Cu/C@MoS_2_ is obviously improved, since the values of ε and ε″of Cu/C@MoS_2_ increase with increasing content of PDA (Fig.. 11h). As shown in Fig. 11i, Cu/C@MoS_2_ 2.0 sample possesses excellent EMW absorption performance. For the sample, its RL value reaches − 48.22 dB (thickness = 2.5 mm, f_E_ = 4.8 GHz), and the widest effective absorption bandwidth reaches 7.12 GHz (thickness = 3.1mm). When the PDA used in the synthesis process is insufficient, the minimum RL value was only -6.75 (for sample Cu/C@MoS_2_ 0.5), meaning that the synergistic effect significantly improves the polarization loss. The EMW attenuation of Cu/C@MoS_2_ is higher than that of C@MoS_2_ and Cu@MoS_2_ which is nearly 266.7% and 222.2%, respectively.

### Construction of Hybrid Heterostructures

Ultrathin-layer structures of 2D TMDs have garnered significant interest owing to their large specific surface area and unique electronic properties. Notably, MoS_2_ nanosheets obtained through the top to down stripping method have been found to possess notable dielectric and microwave absorption characteristics, primarily attributed to the dipole polarization arising from the presence of defects such as Mo and S vacancies. Through precise modulation of the crystal structure or layer number, it is feasible to alter the electronic properties of TMDs from metallic to semiconducting, further demonstrating their great potential for microwave absorption applications. However, the low conductivity of 2D TMDs may not allow for optimal impedance matching. It is challenging to achieve desirable microwave absorption properties for single-component materials without coupling to additional dielectric or magnetic materials. Therefore, the introduction of the second component in TMDs-based absorbers becomes crucial for enhancing their EMW absorption properties. Recently, the phenomenon of synergistic loss has garnered considerable attention, particularly concerning the synergistic losses exhibited by dielectric-dielectric and dielectric-magnetic interactions in transition metal disulfide compounds.

The loss mechanisms of EMA materials can be classified into three main categories: magnetic loss, resistance loss, and dielectric loss. Magnetic loss involves various mechanisms such as hysteresis loss, domain wall resonance loss, ferromagnetic resonance loss, and eddy current loss, all of which contribute to the absorption and attenuation of electromagnetic waves. EMA materials exhibiting magnetic loss primarily include ferrites, carbonyl metal powders, and other magnetic materials. Resistance loss arises from the interaction between materials and the applied electric field. EMA materials demonstrating resistance loss are typically carbon black, graphite, and similar conductive materials. Dielectric loss, on the other hand, occurs through electron polarization, molecular polarization, or interface polarization, allowing for the absorption of electromagnetic waves. EMA materials with dielectric loss encompass compounds such as barium titanate and other dielectric materials.

#### Dielectric-Dielectric Hybridization

##### Carbon Materials

**Fig. 12:**
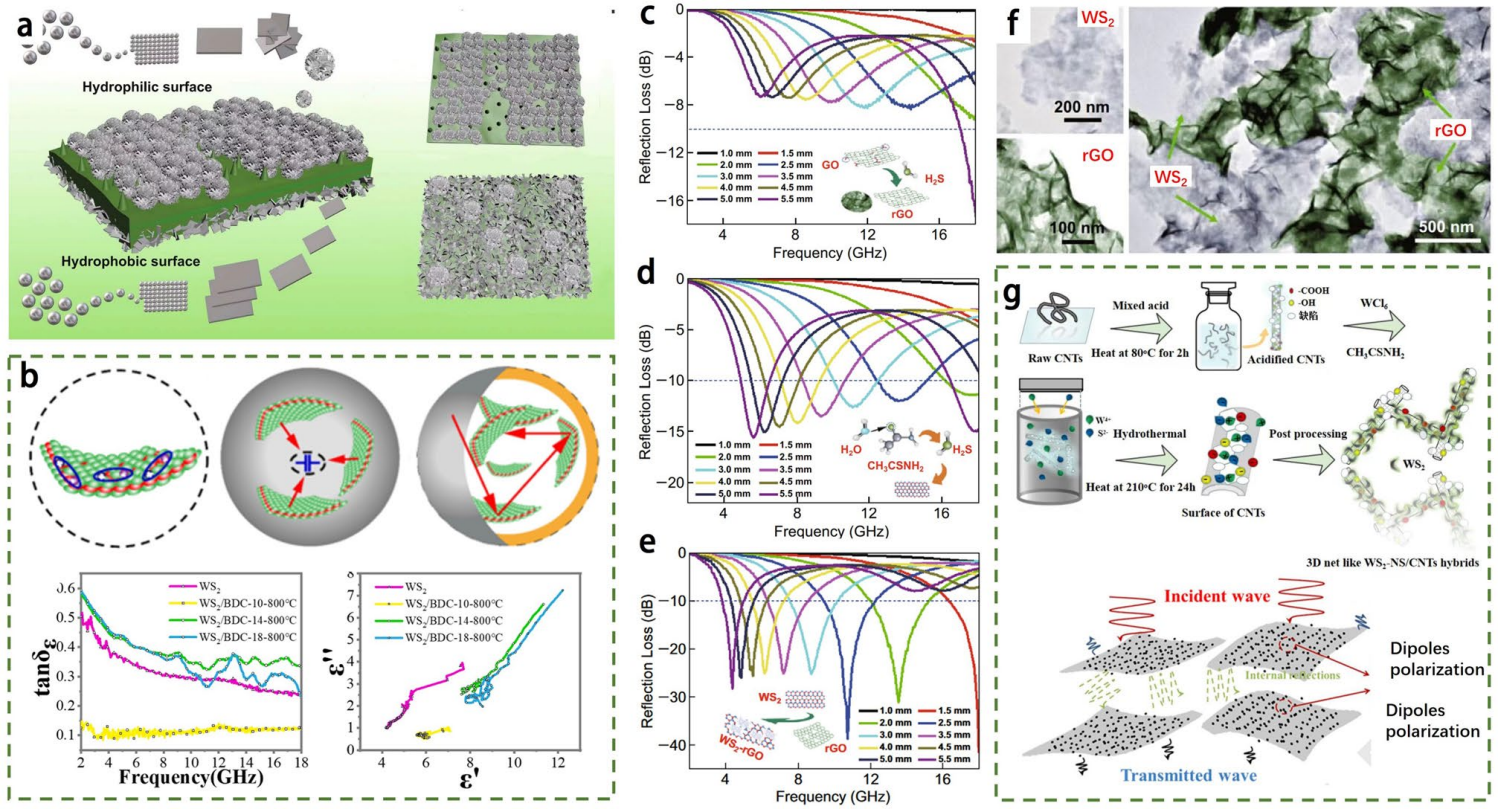
**a** Schematic illustration of the formation process of Janus-like structures. Adapted with permission [95], Copyright 2020 Springer. **b** Schematic illustration of the EMW absorption mechanisms and dielectric properties of WS_2_@BDC composites. Reproduced with permission [96], Copyright 2021 Elsevier B.V. Reflection loss profiles of **c** pure rGO, **d** pure WS_2_ nanosheet, and **e** WS_2_–rGO heterostructure nanosheet powders. **f** TEM images of WS_2_, rGO, WS_2_–rGO, HRTEM image of WS_2_–rGO heterostructure nanosheets. Adapted with permission [9], Copyright 2019 Springer. **g** Schematic illustration of the synthesis process of WS_2_-NS/CNTs hybrids. Adapted with permission [103], Copyright 2020 Elsevier B.V

It has been widely focused and researched that pure carbon-based nanostructures are the most typical dielectric electromagnetic wave attenuating absorbers. It mainly includes graphene, reduced graphene oxide, carbon nanotubes, carbon fibers, and so on. However, bare carbon-based materials often have high conductivity, which causes interface reflection with impedance mismatch in free space. If they are used alone, due to the weak magnetic loss, the absorption intensity does not meet the requirements on effective absorbers, although they may have advantages of low density, excellent dielectric constant, and 2D structure with a large area. Since it is difficult for bare carbon-based materials to achieve excellent electromagnetic wave absorption performance, several researchers synthesize the carbon-based nanostructures containing TMDs and generate specific morphologies, significantly enhancing their wave-absorbing properties. Qi et al. [86] produced MoS_2_/C by hydrothermal reactions. MoS_2_/C possesses excellent dielectric and EMW absorption performance owing to the hybridization with low graphitized carbon, which facilitates electron transport and dielectric polarization. The optimum reflection loss of the composite reaches -44.67dB at a thickness of 1.4 mm with an effective absorption bandwidth of 3.32 GHz at a loading of 30 wt%. With the aim of further optimizing the dielectric properties and improving the reflection loss, Man et al. [93] prepares MoS_2_@HCS hybrids by encapsulating MoS_2_ nanosheets inside hollow carbon spheres via a facile hydrothermal synthesis route. The obtained hybrids exhibited excellent absorptive properties ascribed to the synergistic effect between MoS_2_ and HCSs, which enables rapid electron transport, compensates for the low conductivity of MoS_2_ and MoS_2_ nanosheets, and exposes an adequate number of active sites for polarization and multiple reflections. Similarly, Within the research of Zhou et al. [94], hollow carbon (MHCS) and stacked MoS_2_ nanosheets were composited together to form core–shell nanosphere structures, where the former acts as the core and the latter as the shell. It was found that controlling the thickness of the shells and graphitization of the cores could achieve the modulation of the wave-absorbing properties. The EAB of the hybrids reaches up to 6.21 GHz at a thickness of 2.1 mm. Wei et al. [95] synthesized lotus-derived gradient layered porous C/MoS_2_ morphogenetic composites with broadband and tunable electromagnetic absorption properties. The existence of numerous heterogeneous interfaces between MoS_2_ and C not only enhances the interfacial polarization but also alters the material dielectric constant and optimizes the impedance matching, as shown in Fig. 12a. Cao et al. [96] stacked WS_2_ nanosheets onto the surface of biomass-derived carbon (BDC) via a hydrothermal synthesis route and subsequent carbonization. Compared with pure MoS_2_, the EMW absorption of the obtained composite was significantly enhanced and the effective absorption band was shifted to the lower-frequency region. In addition, electromagnetic parameters and EMW absorption properties in the lower frequency band could be tuned by adjusting the annealing temperatures, as shown in Fig. 12b. They also prepared the VS_2_/ biomass-based GDC with the same preparation method [91]. The loading of VS_2_ nanosheets onto the GDC surface not only enhances the conductive loss, dipole polarization, and interfacial polarization of the pristine material, but also generates multiple reflections and scattering of EMW inside them.

Graphene (GN), as a classical 2D material, has excellent electrical conductivity and extremely high carrier mobility, which is an excellent candidate of electromagnetic wave absorbers. Liu et al. [97] synthesized 2D MoS_2_/GN composites via a simple one-step solvothermal method, achieving effective absorption in Ku band. The optimal reflection loss value at 16.1 GHz is -41.9 dB for a thickness of 2.4 mm, and the RL value exceeds -10 dB across the Ku band (12.2–18.0 GHz) for a thickness of 2.6–3.0 mm. On top of this, Zheng et al. [98] prepared MoS_2_/GN composites with numerous heterogeneous interfaces via a hydrothermal synthesis route. It was found that the hybrids achieved the minimum RL of -55.3 dB at 12.8 GHz with a loading of 20 wt% at a thickness of only 1.6 mm. Similarly, Lu et al. [99] fabricated the three-dimension layered expanded graphite/MoS_2_ nanosheets hybrids through a facile in-situ self-assembling route, which exhibited excellent wave-absorbing performance at only 7 wt% loading. It achieves excellent wave-absorbing performance at only 7 wt% loading. In particular, the optimum reflection loss and the effective absorption band of the composites reached -52.3 dB and 4.1 GHz, respectively, at a thickness of 1.6 mm.

Numerous studies have shown that reduced graphene oxide (RGO) also exhibits EMA performance. Cao et al. [100] produced MoS_2_/RGO hybrid with numerous heterogeneous structure, which exhibits excellent EMA characteristic and broadened EAB through facial hydrothermal synthesis route. In MoS_2_/RGO hybrid, the heterogeneous interface introduced by RGO enhanced the dielectric loss, which increases the EMA performance and broadens the EAB. Similarly, Li et al. [101] prepared hierarchical 2D hybrid containing MoS_2_ nanosheets (NMS) and RGO through a facial intermediate reduction mixing process under different volume ratios. The optimal reflection loss value for NMS/RGO-5:1 loaded with 20 wt% at 2.2 mm is -55 dB. With in the research of Cheng et al. [9], they synthesized WS_2_/RGO nanosheets with plenty of heterogeneous interface through facial hydrothermal synthesis route. Notably, the minimum RL value of the WS_2_-RGO composite reached -41.5 dB with 40 wt% of sample loading while the thickness is 2.7 mm, the EAB of the hybrid is up to 13.62 GHz (4.38–18 GHz), which are much better than bare RGO or MoS_2_, as shown in Fig. 12c-f. The synergistic effect induced by the dielectric-dielectric heterojunction can be employed to explain the dramatic increase in EMW performance.

Single-walled carbon nanotubes (SWCNTs) have a strong dielectric loss due to both high conductivity and a large number of dangling bonds. In addition, the large specific surface area and high aspect ratio enable them to have a great potential for applications in the field of electromagnetic wave absorption. Li et al. [102] produced 1T-WS_2_/SWCNTs hybrids through facial hydrothermal method. It was found that the electromagnetic wave absorption properties could be modulated by controlling the ratio of WS_2_ and SWCNTs. When both are added in equal amounts, the hybrids exhibit the best wave absorption performance, with the EAB varying in the range of 3.0–18.0 GHz during the thickness reduction from 5.0 to 1.0 mm, in addition to the optimal RL value amounting to as high as -66 dB at 8.3 GHz for a thickness of 2.2 mm. On top of this, Cao et al. [103] successfully synthesized ultrathin WS_2_ nanosheet(WS_2_-NS)/ CNTs hybrids using a facial hydrothermal synthesis route. WS_2_-NS is evenly distributed on the surface of CNTs and formed a remarkable three-dimensional (3D) heterogeneous structure (Fig. 12g). It is found that the wave-absorbing properties of the obtained hybrids are highly related to the large number of heterojunction interfaces formed inside the materials, due to the fact that the EMW absorbing properties of the hydrothermally synthesized hybrids are much stronger compared to those of the products obtained from a simple mixing of WS_2_-NS and CNTs by ultrasonication. In the former case, a strong interfacial polarization can be generated under the alternating electromagnetic field due to numerous heterojunction interfaces inside the hybrids, and the resulting dipole polarization and capacitor-like structure can change the electrical properties and greatly enhance the EMW absorbing properties. CF is a material with excellent mechanical strength, and hence it has a wide range of applications in the aerospace industry. However, the disadvantage of impedance mismatch has significantly constrained its development in the field of EMW absorption. Taking the advantage of 1T/2H MoS_2_, Huang et al. [18] designed a EMW flexible materials through growing the 1T/2H MoS_2_ on the surface of CF, and the hybrids exhibited outstanding EMA performance. Particularly, when the filler loading of absorber is only 5%, the optimal reflection loss of CF@1T/2H MoS_2_ was up to − 43 dB at 13.4 GHz with the 2.7 mm thickness.

##### MXene

Since their initial discovery in 2011 by Gogotsi, Ti_3_C_2_T_x_ (where T_x_ represents functional groups such as = O, -OH, and -F) has garnered significant attention as the most extensively studied and representative member of the MXene family. Ti_3_C_2_T_x_ was obtained through the etching of Ti_3_AlC_2_, and its unique characteristics have sparked widespread interest in various research endeavors. The field of microwave absorption, in particular, has been greatly intrigued by its properties [104]. The pure Ti_3_C_2_T_x_ has a narrow layer spacing, but its conductivity is weak and it has limited loss mechanisms and inadequate impedance matching. As a result, its narrow bandwidth limits its further improvement as an EMA material. In a study conducted by Che et al. [105], MXene/MoS_2_ heterostructures with excellent conductivity were successfully prepared through facial hydrothermal synthesis route. The resulting heterostructure exhibited remarkable microwave absorption capabilities, with optimal RL value of -46.72 dB at 2 mm thickness. In comparison, the individual MXene and MoS_2_ materials showed lower absorption performance. Additionally, the researchers visually demonstrated, using electron holography, that an increase in the charge density distribution significantly contributed to the dielectric loss mechanism. Zhang et al. [106] produced MXene/MoS_2_ microspheres through sonication spray self-assembly method. At a thickness of merely 2.5 mm, the material demonstrated an impressive optimal reflection loss of -51.21 dB at 10.4 GHz. Furthermore, an EAB of 4.4 GHz was obtained at 1.6 mm. In another case, through a hydrothermal reaction, Liu et al. [107] successfully synthesized a Ti_3_C_2_Tx/WS_2_ composite, which showcased outstanding electromagnetic absorption performance. The composite exhibited an impressive minimum reflection loss value of -61.06 dB and possessed a broad effective absorption bandwidth of 6.5 GHz.

##### Conductive Polymers

Although conductive polymers have excellent dielectric properties and tunable conductivity as well as low density and diverse molecular structures, their EAB is too narrow in practical applications if they are used as EMA materials. However, they have emerged as ideal candidates for EMA composite materialss. Among them, polyaniline (PANI) has garnered significant attention in recent years. This is primarily due to its straightforward synthesis route, favorable chemical redox properties, robust chemical stability, and cost-effectiveness. In a study conducted by Zhang et al. [108], PANI nanoneedles (PANI-NDs) were grown on a MoS_2_ nanosheet matrix through an in-situ oxidative polymerization process. The resulting MoS_2_/PANI-NDs arrays showcased exceptional electromagnetic wave absorption properties. With a thickness of 1.6 mm, these arrays achieved a remarkable maximum reflection loss value of -44.8 dB at 14.5 GHz. The observed broad EAB, characterized by RL values below -10 dB, can be attributed to the synergistic effect between PANI-NDs and MoS_2_ nanosheets within the composite structure. In a related study by Liu et al. [109], MoS_2_-PANI nanocomposites were synthesized, demonstrating remarkable microwave absorption properties. Particularly, when the ratio of MoS_2_: PANI was balanced at 1:1, the nanocomposite displayed an impressive reflection loss value of -59.78 dB at 8.08 GHz. Moreover, at a thickness of 3.44 mm, the nanocomposite showcased an effective bandwidth of 3.12 GHz, further highlighting its excellent microwave absorption performance. Due to its high electrical conductivity, lightweight nature, convenient synthesis route, and promising physicochemical properties, polycrystalline polypyrrole (PPy) is regarded as a promising material for enhancing the EMA characteristics of MoS_2_-based composites. PPy serves as a competent candidate for optimizing the EMA parameters, contributing to the overall performance improvement of these composites. In an exemplary study conducted by An et al. [110], lightweight, low-cost, and high-performance PPy@MoS_2_ composite absorbers were synthesized, with hierarchical heterostructures through a combination of chemical oxidative polymerization and hydrothermal processes. Notably, the PPy@MoS_2_ composite demonstrated an impressive wide bandwidth (RL < -10 dB) of 6.4 GHz at a thickness of 2.5 mm. Additionally, the composite exhibited an optimum reflection loss value of -49.1 dB at 6.1 GHz, further emphasizing its exceptional performance as an electromagnetic absorber.

#### Dielectric-Magnetic Hybridization

**Fig. 13:**
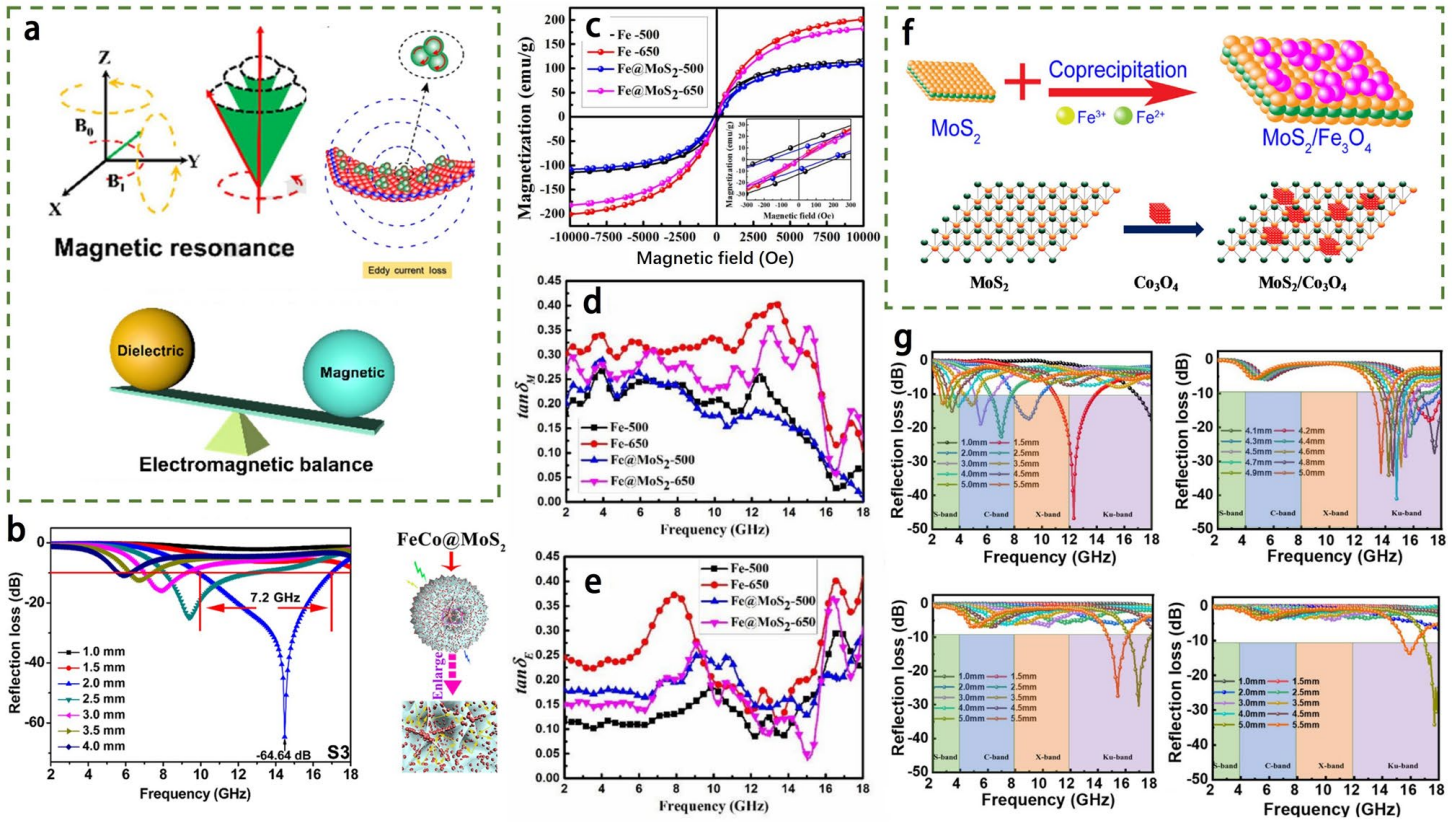
**a** Schematic illustration of dielectric-magnetic junctions for electromagnetic balance. **b** Reflection loss calculated and morphology of the FeCo@MoS_2_ nanoflowers. Adapted with permission [113], Copyright 2018 American Chemical Society. **c** Magnetic hysteresis loops of Fe-500, Fe-650, Fe@MoS_2_-500 and Fe@MoS_2_-650 at room temperature. **d** magnetic loss tangent and **e** dielectric loss tangent of Fe-500, Fe-650, Fe@MoS_2_-500 and Fe@MoS_2_-650. Adapted with permission [112], Copyright 2018 Elsevier B.V. **f** Synthesis routes for the MoS_2_/Fe_3_O_4_ hybrids and MoS_2_/Co_3_O_4_ hybrids. Adapted with permission [100, 115], Copyright 2018 Elsevier B.V.**;** Copyright 2020 Elsevier B.V. **g** RL plots of VS_2_/NiO samples with different hybrid ratios. Adapted with permission [7], Copyright 2022 Springer

The unique electronic structure of 2D TMDs has been proven to grant them excellent electromagnetic wave absorption properties. However, the intrinsic weak impedance matching and sole reliance on the dielectric loss mechanism limit the high-frequency electromagnetic absorption capabilities of TMD-based electromagnetic absorption materials. To address this challenge, various experimental methods have been developed to synthesize dielectric-magnetic junctions, aiming to achieve electromagnetic balance (as depicted in Fig. 13a). By combining magnetic metallic materials with dielectric-loss materials, these composites exploit the synergistic effect between dielectric and magnetic losses while optimizing impedance matching. This approach holds the potential to achieve effective microwave absorption in TMD-based magnetic EMA materials. The development of TMD-based magnetic EMA materials is of significant scientific and engineering importance, as it enables the construction of materials that can effectively absorb and manipulate electromagnetic waves in various applications.

##### Magnetic Metal and Their Alloys

Magnetic metals and their alloys exhibit favorable electromagnetic wave absorption properties due to their high saturation magnetization and permeability. However, the magnetic permeability of these materials is typically constrained by the Snoek limit in the gigahertz range. To overcome this limitation, extensive efforts have been dedicated to the development of composites that combine magnetic metals or their alloys with dielectric materials. In a study by Zou et al. [111], Ni/MoS_2_ nanocomposites were prepared by cladding magnetic Ni nanoparticles onto MoS_2_ micrometer slices. The resulting Ni/MoS_2_ nanocomposites exhibited enhanced electromagnetic characteristics, with optimal reflection loss value of -55 dB when loaded with 60 wt%. The EAB reached up to 4.0 GHz (10.8–14.8 GHz) at a matched thickness of 1.5 mm. Similarly, He et al. [112] successfully fabricated porous coin-shaped Fe@MoS_2_ hybrid through a solvothermal synthesis route, followed by hydrogen reduction at high temperatures. The Fe@MoS_2_-500 hybrid demonstrated a minimum reflection loss of approximately -37.02 dB at an absorber thickness of 2.0 mm. Moreover, the composite exhibited a maximum EAB ranging from 11.13 to 15.86 GHz, spanning approximately 4.73 GHz. The effective microwave absorption band of the Fe@MoS_2_-650 composites shifted to lower frequencies when the calcination temperature was increased to 650 °C, while maintaining a constant absorber thickness. This shift was attributed to improved impedance matching and the synergistic effect between dielectric loss and magnetic loss. In another study by Wu et al. [113], FeCo/MoS_2_ nanoflowers were fabricated, showcasing exceptional EMW absorption properties. The combined magnetic and dielectric losses collaborate to enhance impedance matching and absorption performance in a synergistic manner. The interaction between these losses leads to improved absorption characteristics by effectively attenuating and dissipating electromagnetic energy, with a reflection loss of -64.64 dB at 14.4 GHz. The composite also exhibited a wide frequency range (7.2 GHz) for RL < -10 dB, as shown in Fig. 13b.

##### Metal Oxides

Extensive research has been conducted on metal oxide nanoparticles such as Fe_3_O_4_, Co_3_O_4_, and NiO to enhance the dielectric and magnetic losses of EMA materials. These nanoparticles possess desirable properties including high saturation magnetization, broad absorption spectra, high permeability, abundant active sites, and selectivity. Fe_3_O_4_, for instance, exhibits a large magnetic moment, good electrical conductivity, low dielectric constant, and high Curie temperature. These characteristics contribute to the reduction of magnetic and dielectric losses, making Fe_3_O_4_ a representative material for high-performance electromagnetic wave absorption. The effective integration of magnetic Fe_3_O_4_ nanoparticles with transition metal dichalcogenides is considered a promising and necessary approach in achieving enhanced EMA performance. In a pioneering study, Cao et al. [100] utilized a hydrothermal and subsequent co-precipitation method to prepare a MoS_2_/Fe_3_O_4_ hybrid with heterostructures. The EMA performance of this hybrid material was investigated for the first time, as depicted in Fig. 13f. When the loading is 40%, MoS_2_/Fe_3_O_4_ hybrid achieved the optimal RL value of -42.57 dB with the thickness of 3.25 mm. Impressively, the composite exhibited a wide bandwidth, with reflection loss values below -10 dB spanning 12.73 GHz (ranging from 5.27 to 18 GHz). Similarly, Wu et al. [114] employed hydrothermal synthesis route to synthesize hierarchical flower-like Fe_3_O_4_/MoS_2_ hybrid. By adjusting the Fe_3_O_4_ content, various morphologies of Fe_3_O_4_/MoS_2_ hybrid were obtained. These hybrids exhibited enhanced electromagnetic wave absorption performance, with a minimum reflection loss of -64 dB with the thickness of 1.7 mm. Moreover, at an ultrathin thickness of 2.0 mm, the composites exhibited an effective absorption bandwidth of 6.1 GHz. These improved absorption properties were attributed to the good impedance matching and the dielectric and magnetic loss characteristics of the composites.

Co_3_O_4_ nanoparticles (Co_3_O_4_-NPS) have been recognized for their unique electromagnetic properties and semiconducting properties, making them effective in improving the EMA performance. Cao et al. [115] employed a facile hydrothermal synthesis route to implant Co_3_O_4_-NPS on MoS_2_ nanosheets to enhance the EMA performance, as depicted in Fig. 13f. With a Co_3_O_4_ loading of 20 wt% and a sample thickness of 4.0 mm, the MoS_2_/Co_3_O_4_ hybrid demonstrated an impressive minimal RL value of -43.56 dB at 6.96 GHz. Moreover, the hybrid material exhibited a wide bandwidth of 4.76 GHz (ranging from 13.24 to 18 GHz) with RL values below 10 dB at an absorber thickness of 2.0 mm. The outstanding performance of this hybrid can be attributed to the effective adjustment of impedance matching and the synergistic enhancement of magnetic and dielectric losses achieved through the incorporation of Co_3_O_4_ nanoparticles within the MoS_2_ nanosheets. Moreover, Cao and colleagues [116] successfully fabricated hybrid nanosheets composed of Co_3_O_4_ and WS_2_ with heterostructures, combining the magnetic and dielectric properties of Co_3_O_4_ and WS_2_, respectively. The resulting Co_3_O_4_-WS_2_ hybrid nanosheets exhibited electromagnetic properties resulting from the synergistic combination of these two components. Leveraging the electromagnetic synergy effect, the hybrid nanosheets achieved an impressive maximum RL value of -61.1 dB at a thickness of 1.9 mm, accompanied by a bandwidth exceeding 5 GHz. Additionally, VS_2_ has emerged as an attractive candidate for low-frequency EMA materials due to its remarkable features, including a large specific surface area, unique electronic structure, and propensity to form heterostructures with tailored properties. Cao et al. successfully fabricated hierarchical heterostructures by incorporating Co_3_O_4_ nanoparticles into VS_2_ nanosheets. The uniform dispersion of Co_3_O_4_ nanoparticles played a pivotal role in establishing a electromagnetic coupling network, thereby significantly improving the loss characteristics of EMA materials [117].

Nickel oxide (NiO) nanoparticles possess distinctive physical and chemical properties, including high saturation magnetization, permeability, abundant active sites, and size effects. These properties make them widely employed in enhancing the electromagnetic characteristics of dielectric EMA materials. Cao and colleagues [59] successfully synthesized WS_2_/NiO hybrids with heterostructures through a straightforward hydrothermal synthesis route. The incorporation of NiO led to a significant improvement in the EMA performance of the hybrids. Remarkably, an RLmin value as low as -53.31 dB was achieved with the thickness of 4.30 mm. Furthermore, the hybrids demonstrated a bandwidth of 13.46 GHz (ranging from 4.54 to 18 GHz) with RL values below -10 dB for absorber thicknesses between 3.5 and 5.5 mm. Similarly, in another study [7], Cao and his team prepared VS_2_/NiO composites using a hydrothermal synthesis route. The minimum reflection loss value reached -58.98 dB at 12.32 GHz for a sample with a thickness of 1.48 mm when the NiO loading in the VS_2_/NiO hybrid material was 20%. Additionally, the sample exhibited a bandwidth of 3.5 GHz with RL values below -10 dB when the thickness was reduced to 1.2 mm, as depicted in Fig. 13g. These results demonstrate that the significant enhancement in microwave absorption performance can be attributed to the satisfactory dielectric and magnetic loss capabilities, good impedance matching, and the unique laminated structure of the materials.

The fabrication of hybrid core–shell microspheres combining MoS_2_ with various magnetic multiferroics materials has emerged as a promising approach to enhance EMA performance. Several studies have focused on the synthesis and characterization of these hybrid structures, revealing impressive microwave absorption capabilities. In a facile hydrothermal synthesis route, Lin et al. [118] successfully synthesized flower-like core–shell microspheres consisting of MoS_2_@Bi_2_Fe_4_O_9_ microspheres (MPs). By optimizing the dosage of Bi_2_Fe_4_O_9_ MPs, the MoS_2_@Bi_2_Fe_4_O_9_ MPs achieved a remarkable maximum RL value of -52.3 dB at 12.4 GHz, with a thickness of 2.8 mm. Moreover, these hybrid microspheres demonstrated an effective bandwidth (RL ≤ -10 dB) as high as 5.0 GHz, spanning from 10.4 to 15.4 GHz. BaFe_12_O_19_, classified as an M-type hexagonal ferrite material with high complex permeability, faces limitations due to its low dielectric constant and narrow bandwidth when seeking further enhancements in microwave absorption. To address this challenge, Lin et al. successfully prepared a novel core–shell composite of BaFe_12_O_19_@MoS_2_ using a hydrothermal method. Notably, the BaFe_12_O_19_@MoS_2_ composite exhibited an impressive maximum reflection loss of -61.0 dB with the thickness of 1.7 mm. Additionally, the composite showcased an EAB of 4.4 GHz when thiourea was added at a concentration of 28 mmol [119]. Qi et al. successfully constructed and synthesized core–shell nanocomposites of Co_0.6_Fe_2.4_O_4_@MoS_2_, showcasing remarkable microwave absorption properties [120]. Notably, the optimized RL value reached an impressive -79.9 dB at 11.2 GHz for a thickness of 2.73 mm. Furthermore, for thicknesses of 2.34 and 2.98 mm, the nanocomposites exhibited an EAB of 5.96 GHz. Similarly, Wang et al. [121] successfully developed a novel microwave absorber based on a 3D nested core–shell structure of CoFe_2_O_4_@1T/2H-MoS_2_. This structure involved embedding CoFe_2_O_4_ nanospheres into the holes of 3D nested 1T/2H multiphase MoS_2_. Remarkably, the synthetic composite exhibited an optimal reflection loss of -68.5 dB at 13.2 GHz, with a thickness of 1.81 mm. Additionally, a composite with a thickness of 1.6 mm achieved a wide EAB of 4.56 GHz, ranging from 13.2 to 17.76 GHz. Moreover, the overall EAB covered a broad range of 14.5 GHz, spanning from 3.5 to 18.0 GHz, which accounted for more than 90% of the measurement frequency range. The exceptional microwave absorption performance of this composite can be attributed to its unique structural design, with magnetic CoFe_2_O_4_ nanorods in the core and dielectric nested 1T/2H-MoS_2_ in the shell, along with the proper impedance matching between them. Notably, ZnFe_2_O_4_ and CuFe_2_O_4_ are also noteworthy members of the multiferroics family. In a facile hydrothermal synthesis route, Wang et al.[122] synthesized a novel flower-like core–shell composite of ZnFe_2_O_4_@MoS_2_. When the filler content reached 20 wt%, the ZnFe_2_O_4_@MoS_2_ composite exhibited an optimal reflection loss of -61.8 dB at 9.5 GHz. Furthermore, the corresponding effective bandwidth (RL > -10 dB) extended over 5.8 GHz, ranging from 7.2 to 13 GHz. This impressive EMA performance can be attributed to several factors, including the porous core–shell structure, strong interfacial polarization, multiple reflections, matched impedance, and the synergistic effect between the ZnFe_2_O_4_ core and the MoS_2_ shell. In a study conducted by Wu et al. [123], the synthesis of a CuFe_2_O_4_/MoS_2_ composite was achieved through solvothermal synthesis route. The findings indicate that the CuFe_2_O_4_/MoS_2_ composites exhibit exceptional EMA absorption capabilities. Specifically, at a matching thickness of 2.7 mm, the composite demonstrates a remarkable RL_min_ value of -49.43 dB at 10.4 GHz. Furthermore, the composite achieves an impressive maximum effective absorption bandwidth of 8.16 GHz at a thickness of 2.3 mm. The introduction of magnetic particles effectively balances the high dielectric constant of MoS_2_, leading to excellent impedance matching performance.

By fabricating these composites, researchers aim to enhance the overall electromagnetic absorption performance by leveraging the unique properties of both magnetic and dielectric components. The incorporation of dielectric materials helps overcome the limitations of magnetic metals in the gigahertz range, thereby enabling improved electromagnetic wave absorption characteristics. This approach has shown promise in enhancing the absorption capabilities of these composite materials.

#### Ternary Heterojunctions

As mentioned above, the compounds containing TMDs and dielectric and magnetic materials have significantly improved microwave absorption performance compared to bare TMDs, due to the synergistic effect of dielectric-dielectric and dielectric-magnetic in the compounds. Accordingly, multi-component (triple or even quadruple) TMDs-based hybrids should have superior microwave absorption properties, whereas the quadruple hybrids are more difficult to achieve large-scale application due to their complicated preparation process. A large number of studies have been carried out on triple TMDs-based composite absorbers. Most of the synthesized hybrids are realized in the semiconductor-conductor-magnetic material (SCM) mode, which can make full use of dielectric, conductivity and magnetic losses to enhance the attenuation of electromagnetic waves within the materials, such as FeNi_3_@RGO/MoS_2_ [124], PPy/LiFe_5_O_8_/MoS_2_ [125], ZnCo@C@1T-2H-MoS_2_ [126], Co@NC@MoS_2_ [127], MoS_2_@PPy@Fe_3_O_4_ [128], and rGO@Ni-doped-MoS_2_ [129]. In addition, using one-pot hydrothermal method, Wang et al. [130] synthesized MoS_2_/Fe_3_O_4_/graphene composites. The results demonstrated that these composites exhibited remarkable electromagnetic absorption properties, with optimal reflection loss value of -45.8 dB at 5.9 GHz with the thickness of 2.5 mm. Additionally, the EAB, defined by RL values below -10 dB, covered a wide range of almost 7.4 GHz (4.6–9.7 GHz, 15.7–18 GHz) at a thickness of 2.5 mm. The exceptional EMW absorption performance of the composites can be attributed to their suitable impedance matching and multi-polarization characteristics. Ning et al. [131] synthesized Fe_3_O_4_@NC@MoS_2_ nanostructures using a simple wet chemical route. These nanostructures exhibited satisfactory electromagnetic wave attenuation properties across a frequency range of 2 − 18 GHz, with a reflection loss value of -68.9 dB and an effective absorption bandwidth of 5.2 GHz. The enhanced microwave absorption is attributed to tailored synergistic effects between dielectric and magnetic losses, as well as the introduced interfacial polarization in the hybrid. In another study, Qi et al. [132] deposited Fe_3_O_4_ nanoparticles onto the surface of few-layer MoS_2_ nanosheets through hydrothermal synthesis route, followed by a carbonation process through CVD. Zhang et al. [133] successfully fabricated polyaniline@MoS_2_ (PANI@MoS_2_) and PANI@MoS_2_@Fe_3_O_4_ with layered structures for microwave absorption. The results demonstrated that optimal impedance matching and simultaneous suppression of eddy currents were achieved only with the best Fe_3_O_4_ nanoparticle coating. Liu et al. [134] fabricated ternary hybrids of Co_9_S_8_/CNTs/MoS_2_ for absorbing electromagnetic waves, utilizing the assembly of MoS_2_ nanosheets and Co_9_S_8_ particles onto interconnected microcages derived from MOF. The CNT porous microcages, with appropriate electrical conductivity, promoted conduction loss and facilitated optimal impedance matching. Furthermore, the arrangement of MoS_2_ nanosheets on folded surfaces contributed to inherent polarization loss and increased the scattering area for electromagnetic waves. The dispersed Co_9_S_8_ particles functioned as dielectric additives, enhancing the effect of interfacial polarization. Similarly, MoS_2_@Fe_3_O_4_-GNs ternary composites with hollow spherical structures were synthesized using hydrothermal and solvothermal approach. These composites exhibit not only favorable impedance matching performance but also effective coordination between magnetic and dielectric losses. The successful integration of the hollow core–shell structure of MoS_2_@Fe_3_O_4_ and the hollow core–shell structure of GNs enables outstanding EMA capabilities in the ternary composites [135].

In addition to the semiconductor–semiconductor-magnetic (SCM) configuration of composite, the semiconductor-conductor-conductor (SCC) configurations are often used to design TMDs-based composite absorbing materials. Jia et al. [136] developed a novel multilayer-scale structure absorber composed of NiS/MoS_2_/Ti_3_C_2_T_x_ hybrid materials. This absorber incorporates highly conductive Ti_3_C_2_T_x_ as substrates and TMDs (NiS and MoS_2_) to enhance its EMW absorption capability. The inclusion of TMDs allows for the adjustment of the dielectric parameters of Ti_3_C_2_T_x_, thereby achieving improved impedance matching in free space. Additionally, the presence of NiS and MoS_2_ semiconductors induces an inhomogeneous distribution of space charge when exposed to an alternating electromagnetic field, thereby enhancing the polarization process and resulting in electromagnetic wave attenuation. Liu et al. [137] conducted a study where they fabricated a layered structure of carbon fiber@MXene@MoS_2_ (CMN) to achieve adjustable and efficient EMW absorption properties. The combined effect of increased conductive loss from the MXene sheath and improved impedance matching provided by the outermost MoS_2_ layer effectively regulated and optimized the EMA performance of the CMM composite. The optimized thickness of 3.5 mm resulted in an impressive reflective loss of -61.51 dB, and at a thickness of 2.1 mm, the composite exhibited a maximal effective absorption bandwidth that covered the entire Ku-band at 7.6 GHz. Additionally, with a specific loading of MoS_2_ at the optimized thickness, absorption across the entire X-band could be achieved.

In conclusion, the combination of TMDs with dielectric and magnetic materials has led to significant improvements in microwave absorption performance. Hybrid composites incorporating TMDs in various configurations, such as SCM and SCC, have demonstrated superior absorption capabilities through the synergistic effects of dielectric, conductivity, and magnetic losses. These composites exhibit suitable impedance matching, interfacial polarization, and coordination between magnetic and dielectric properties, resulting in exceptional microwave absorption performance.

## Conclusion and Outlook

In recent years, considerable research efforts have been carried out in the field of electromagnetic wave absorbers based on the VIB- and VB-group TMDs, due to their unique physicochemical properties such as unique phase structure, large specific surface area, etc. In this review, we summarize the EMW absorption and attenuation mechanisms based on those investigations, and describe the microstructural and electrical properties of VIB- and VB-group TMDs as well as the relations between them. We also summarize various routes that TMDs-based absorbers can be synthesized, and those cases of constructing heterojunctions inside the absorbers, with their structural and wave absorbing properties listed in Table 2. In conclusion, TMDs can be equipped with special structures by adjusting and optimizing the synthesis routes; their electromagnetic parameters can be regulated and their impedance matching and electromagnetic loss can be improved to achieve excellent electromagnetic wave absorption. Particularly, the unique phase structure of TMDs can be tuned by controlling the reaction conditions (e.g., for NbS_2_) [16]. In addition, the morphology, defects, and heterojunction of the materials can be optimized by different preparation routes. The direct exfoliation of bulk materials leads to the formation of few- or single-layer TMDs, exposing a larger number of active sites. CVD is widely recognized as an ideal technique for producing high-quality, low-thickness nanostructures with minimal defects on diverse substrates, enabling detailed investigations into the electromagnetic wave absorption mechanisms of TMDs. Hydrothermal or solvothermal methods provide convenient means to prepare TMDs nanomaterials with distinct morphological characteristics, phases, or crystallinity, thereby allowing for finely tuning their EMA performance from multiple perspectives. There are three types of construction of hybrid heterostructures, including the dielectric-dielectric hybridization of carbon, MXene, and conductive polymers, the dielectric-magnetic hybridization of magnetic metal and their alloys and metal oxides, and the ternary heterojunctions. It is evident that TMDs have great application prospects in the field of wave absorption. Nevertheless, there are obstructions and opportunities that may exist in the development of TMDs based microwave absorbing materials, and some unresolved issues and challenges still need to be addressed, which are summarized as follows: (1) Facial synthesis routine: The industrial-scale application of TMDs absorber materials has to be implemented through simplifying their preparation process and reducing their production cost. Some rapid preparation techniques, such as solvothermal, hydrothermal, and microwave synthesis, can significantly reduce the preparation time and boost the processing efficiency. The utilizations of low-cost raw materials and equipment can also help to reduce the production cost. Furthermore, the preparation process can be remarkably simplified if the enhancement of the absorption and broadband performance of TMDs based absorber materials can be realized by composition adjustment. In addition, the modification on the layer number, morphology, size, and other microstructure parameters of TMDs can affect their electromagnetic properties, which are also effective in tuning their EMW absorption effect. The strategies mentioned above are essential for streamlining the preparation process, reducing production costs, and facilitating the large-scale utilization of TMDs based absorber materials.

**Table 2:** Summary of structural engineering and electromagnetic wave absorption performance of TMDs

Materials	Morphology	Type of heterojunction	Filler loading/wt%	RL_min_			EAB		Refs
				dB	GHz	mm	GHz	mm	
*Morphology engineering*									
NbS_2_	hollow-sphere	–	–	−43.85	7.82	3.50	6.43	2.09	[6]
1 T/2H MoS_2_	flower-like	–	60%	−56.32	17.16	1.85	5.88	2.13	[5]
MoS_2_/C	microspheres	D-D	30%	−44.67	–	1.40	3.32	1.40	[72]
MoS_2_	flower-like	–	30%	−39.20	17.60	2.40	7.60	3.00	[73]
VS_2_	nanosheets	–	–	-57.50	4.00	5.75	7.00	–	[74]
WS_2_	nanosheets	–	30%	−63.00	–	2.50	4.60	2.50	[75]
MoS_2_	nanosheets	–	60%	−38.42	–	2.40	4.1	–	[76]
*Phase engineering*									
1 T/2H MoS_2_	rippled	–	–	−45.50	–	–	3.89	–	[9]
1 T/2H MoS_2_	flower-like	–	60%	−63.78	11.12	2.57	5.48	1.93	[7]
VS_2_/GDC	nanosheets	D-D	–	−52.80	12.20	2.70	5.70	2.50	[36]
1 T/2H MoS_2_	–	–	15%	−52.70	17.70	2.60	–	–	[8]
*Hybrid engineering*									
MoS_2_@HCS	hollow-sphere	D-D	60%	−65.00	–	2.00	5.00	–	[79]
C/MoS_2_	hierarchical	D-D	–	−50.10	–	2.40	6.00	2.20	[81]
WS_2_@BDC	–	D-D	–	−51.40	5.52	4.50	–	–	[1]
VS_2_/GDC	–	D-D	–	−52.80	12.20	2.70	5.70	2.50	[36]
MoS_2_/graphene	–	D-D	–	−41.90	16.10	2.40	–	–	[82]
MoS_2_/GN	–	D-D	20%	−55.30	–	1.60	5.60	2.20	[83]
EG/MoS_2_	honeycomb-like	D-D	7%	−52.30	–	1.60	4.10	1.60	[84]
MoS_2_/RGO	–	D-D	40%	−49.41	–	2.52	13.36	2.52	[85]
WS_2_/RGO	–	D-D	40%	−41.50	–	2.70	13.62	2.70	[45]
MoS_2_/RGO	–	D-D	20%	−55.00	–	2.20	6.96	–	[86]
WS_2_/SWCNTs	–	D-D	–	−66.00	8.30	2.20	–	–	[87]
WS_2_-NS/CNTs	–	D-D	–	−51.60	14.80	1.95	5.40	1.95	[88]
CF@MoS_2_	–	D-D	5%	−43.00	13.40	2.70	–	–	[8]
MXene/MoS_2_	–	D-D	–	−46.72	–	2.00	4.32	2.00	[90]
MXene/MoS_2_	–	D-D	30%	−51.21	10.40	2.50	4.40	1.60	[91]
Ti_3_C_2_T_x_/WS_2_	–	D-D	–	−61.06	13.28	–	6.50	–	[92]
MoS_2_/PANI	–	D-D	–	−44.80	14.50	1.60	–	–	[93]
MoS_2_-PANI	–	D-D	–	−59.78	8.08	3.44	3.12	3.44	[94]
PPy@MoS_2_	–	D-D	–	−49.10	6.10	2.50	6.40	2.50	[95]
Ni/MoS_2_	–	D-M	60%	−55.00	–	1.50	4.00	1.50	[96]
Fe@MoS_2_	–	D-M		−37.02		2.00	4.73	2.00	[97]
FeCo/MoS_2_	–	D-M	–	−64.64	14.40	2.00	7.20	2.00	[98]
MoS_2_/Fe_3_O_4_	–	D-M	40%	−42.57	–	3.25	12.73	3.25	[85]
MoS_2_/Fe_3_O_4_	–	D-M	–	−64.00	–	1.70	6.10	2.00	[99]
MoS_2_/Co_3_O_4_	–	D-M	–	−43.56	6.96	4.00	4.76	2.00	[100]
Co_3_O_4_/WS_2_	–	D-M	–	−61.10	–	1.90	5.00	1.90	[101]
VS_2_/Co_3_O_4_	–	D-M	–	−57.96	–	1.57	3.50	1.57	[102]
WS_2_/NiO	–	D-M	–	−53.31	–	4.30	13.46	–	[43]
VS_2_/NiO	–	D-M	–	−58.98	12.32	1.48	3.50	1.20	[103]
MoS_2_@Bi_2_Fe_4_O_9_	core–shell	D-M	–	−52.30	12.40	2.80	5.00	2.80	[104]
BaFe_12_O_19_@MoS_2_	core–shell	D-M	–	−61.00	–	1.70	4.40	1.70	[105]
Co_0.6_Fe_2.4_O_4_@MoS_2_	core–shell	D-M	–	−79.90	11.10	2.73	5.69	2.34	[106]
CoFe_2_O_4_@MoS_2_	core–shell	D-M	–	−68.50	13.20	1.81	4.56	1.60	[107]
ZnFe_2_O_4_@MoS_2_	core–shell	D-M	20%	−61.80	9.50	–	5.80	–	[108]
CuFe_2_O_4_/MoS_2_	–	D-M	–	−49.43	10.40	2.70	8.16	2.30	[109]
FeNi_3_@RGO/MoS_2_	–	Multiple	40%	−30.39	–	2.00	4.72	2.00	[110]
PPy/LiFe_5_O_8_/MoS_2_	coral-like	Multiple	–	−73.25	–	3.07	7.20	3.14	[111]
ZnCo@C@MoS_2_	core–shell	Multiple	–	−35.83	5.83	5.00	4.56	2.00	[112]
Co@NC@MoS_2_	core–shell	Multiple	15%	−61.97	9.20	–	5.60	–	[113]
MoS_2_@PPy@Fe_3_O_4_	–	Multiple	–	−32.00	–	2.00	–	–	[114]
rGO@Ni-MoS_2_	–	Multiple	–	−40.00	–	2.00	–	–	[115]
MoS_2_/Fe_3_O_4_/GN	–	Multiple	–	−45.80	5.90	2.50	7.40	2.50	[116]
Fe_3_O_4_@NC@MoS_2_	dumbbell	Multiple	–	−68.90	–	–	5.20	–	[117]
MoS_2_/Fe_3_O_4_/C	–	Multiple	–	−53.03	14.40	7.86	–	–	[118]
PANI@MoS_2_@Fe_3_O_4_	–	Multiple	–	−49.70	–	1.30	6.48	1.70	[119]
Co_9_S_8_/CNTs/MoS_2_	–	Multiple	9%	−35.40	–	–	8.40	–	[120]
MoS_2_@Fe_3_O_4_-GNs	hollow	Multiple	–	−48.10	10.08	2.60	4.08	1.70	[121]
NiS/MoS_2_/Ti_3_C_2_T_x_	–	Multiple	–	−58.48	–	2.40	5.04	2.10	[122]
CF@MXene@MoS_2_	core–shell	Multiple	–	−61.51	–	3.50	7.60	2.10	[123]

– represents the values are not available, D-D means dielectric-dielectric heterojunction, D-M means dielectric-magnetic heterojunction

(2) Precisely controlled process: The precise control of the preparation of TMDs based absorber materials is one of the key strategies for achieving high-performance absorber materials. Advanced preparation techniques such as chemical vapor deposition and physical vapor deposition can precisely control the composition and morphology of the material at atomic levels, thereby adjusting its electromagnetic properties. By precisely controlling the composition of TMDs, such as layer number, elemental composition, and crystal structure, the electromagnetic wave absorption characteristics can be adjusted to achieve efficient absorption. In addition, precise control of the morphology of TMDs based absorber materials is also an important strategy for achieving high-performance absorber materials. By preparing TMDs absorber materials with hierarchical structures, the material's specific surface area and interfacial reaction can be increased, thereby improving absorption performance and stability. In these structures, the interaction between interfaces at different levels is beneficial to weaken the propagation and reflection of electromagnetic waves, allowing for efficient absorption in a wide frequency band. In addition, it is also an effective strategy that TMDs based absorber materials with porous structures are prepared, which can increase the material's degree of impedance matching, improve the EMW absorption performance and wide-band performance. In those porous structures, the voids can effectively adjust the material's dielectric constant and magnetic permeability, allowing for good impedance matching over a wide frequency band. Moreover, the porous structure also has high specific surface area and porosity, which can increase the reflection and scattering of the absorption surface, thereby improving the absorption performance and stability of EMA materials.

(3) For application: stability and functionality: In practical applications, it is important to consider both the stability and functionality of TMDs based absorber materials. For instance, in the fields of their applications such as electronic devices, aerospace, and aviation, materials with lightweight and flexible properties are required to adapt to complex working environments and service conditions. Moreover, the durability and stability of the materials are critical factors to be addressed in their applications, which necessitate the optimization of the physical and chemical properties of the materials to improve their stability and lifespan. Therefore, in order to achieve the desired performance and meet the practical requirements, it is essential to consider both the stability and functionality of TMDs based absorber materials in their development and application.
